# Quantitative modelling of hip fracture trends in 14 European countries: testing variations of a shared reversal over time

**DOI:** 10.1038/s41598-017-03847-x

**Published:** 2017-06-16

**Authors:** Raquel Lucas, Ana Martins, Milton Severo, Poliana Silva, Teresa Monjardino, Ana Rita Gaio, Cyrus Cooper, Henrique Barros

**Affiliations:** 1 EPIUnit – Institute of Public Health, University of Porto, Porto, Portugal; 2 Department of Clinical Epidemiology, Predictive Medicine and Public Health, University of Porto Medical School, Porto, Portugal; 3 Department of Mathematics, Faculty of Sciences, University of Porto and Centre of Mathematics of the University of Porto, Porto, Portugal; 4 MRC Lifecourse Epidemiology Unit, University of Southampton, Southampton General Hospital, Southampton, UK

## Abstract

Qualitative similarities between hip fracture trends in different countries suggests variations of the same epidemic. We tested a single statistical shape to describe time trends in Europe, while allowing for country-level variability. Using data from 14 countries, we modelled incidence rates over time using linear mixed-effects models, including the fixed effects of calendar year and age. Random effects were tested to quantify country-level variability in background rates, timing of trend reversal and tempo of reversal. Mixture models were applied to identify clusters of countries defined by common behavioural features. A quadratic function of time, with random effects for background rates and timing of trend reversal, adjusted well to the observed data. Predicted trend reversal occurred on average in 1999 in women (peak incidence about 600 per 100 000) and 2000 in men (about 300 per 100 000). Mixture modelling of country-level effects suggested three clusters for women and two for men. In both sexes, Scandinavia showed higher rates but earlier trend reversals, whereas later trend reversals but lower peak incidences were found in Southern Europe and most of Central Europe. Our finding of a similar overall reversal pattern suggests that different countries show variations of a shared hip fracture epidemic.

## Introduction

Due to pathophysiology, prognosis and healthcare implications, age-related hip fractures are socially the most relevant outcome of bone fragility. In Belgium, a country with intermediate incidence, the remaining risk of hip fracture was about 20% in women aged 60^1^ and a 24% functional decline in the year after fracture was estimated^2^. Mortality is also a major concern: in the UK, for instance, one-year case-fatality remained over 20% from 2000 to 2010^3^. In comparative terms, fragility fractures account for more disability-adjusted life years than most common cancers in Europe^4^.

Since hip fractures almost inevitably involve hospital admission, incidence estimated from healthcare is particularly valid to compare the burden of bone fragility between populations and within the same population over time. Some European countries have a long tradition of describing secular trends of hip fractures, and evidence of increasing rates during the 1970s was found in the UK^5^, Sweden^6^, Finland^7^ and the Netherlands^8^. Subsequent increases were seen up to the 1990s in other countries, namely Austria^9^, Denmark^10^, Germany^11, 12^, Iceland^13^, Norway^14^, and Spain^15^. More recently, however, a reversal of age-specific trends was detected, first in the UK^5^ and Scandinavia^7, 14, 16, 17^, and subsequently in many other settings^9, 18–20^. As countries gained improved access to hospital records, publications on the subject grew substantially. Two comprehensive reviews^21, 22^ show that trend reversals in hip fracture incidence are common in European countries, even though the timing has varied between settings^21^. Candidate explanations for trend reversals are multiple, and include planned efforts targeting bone fragility as well as macro-level changes that may have caused decreasing fracture trends as an unpredicted effect^22^.

Qualitative similarities between countries led us to hypothesize that a single shape could describe the hip fracture epidemic in Europe. Specifically, there seems to be considerable country-level evidence that secular trends can be modelled as a quadratic function of time, i.e. a first period of increasing rates but decreasing acceleration followed by a short stagnation with null acceleration and finally a later period of decreasing rates with increasingly negative acceleration. Nevertheless, within a common overall pattern, specific features of trend reversals may differ between countries, namely background fracture rates, timing and tempo of reversal. The hypothesis that different countries have followed different variations of the same hip fracture epidemic is a starting point for testing mechanistic explanations and, ultimately for future policy modelling, as shown by previous successful approaches to other conditions^23^.

In the present work, we aimed to model recent hip fracture secular trends in different European countries and to identify clusters of countries with similar behavioural features over time.

## Methods

### Selection of time series and data extraction

**Figure 1:**
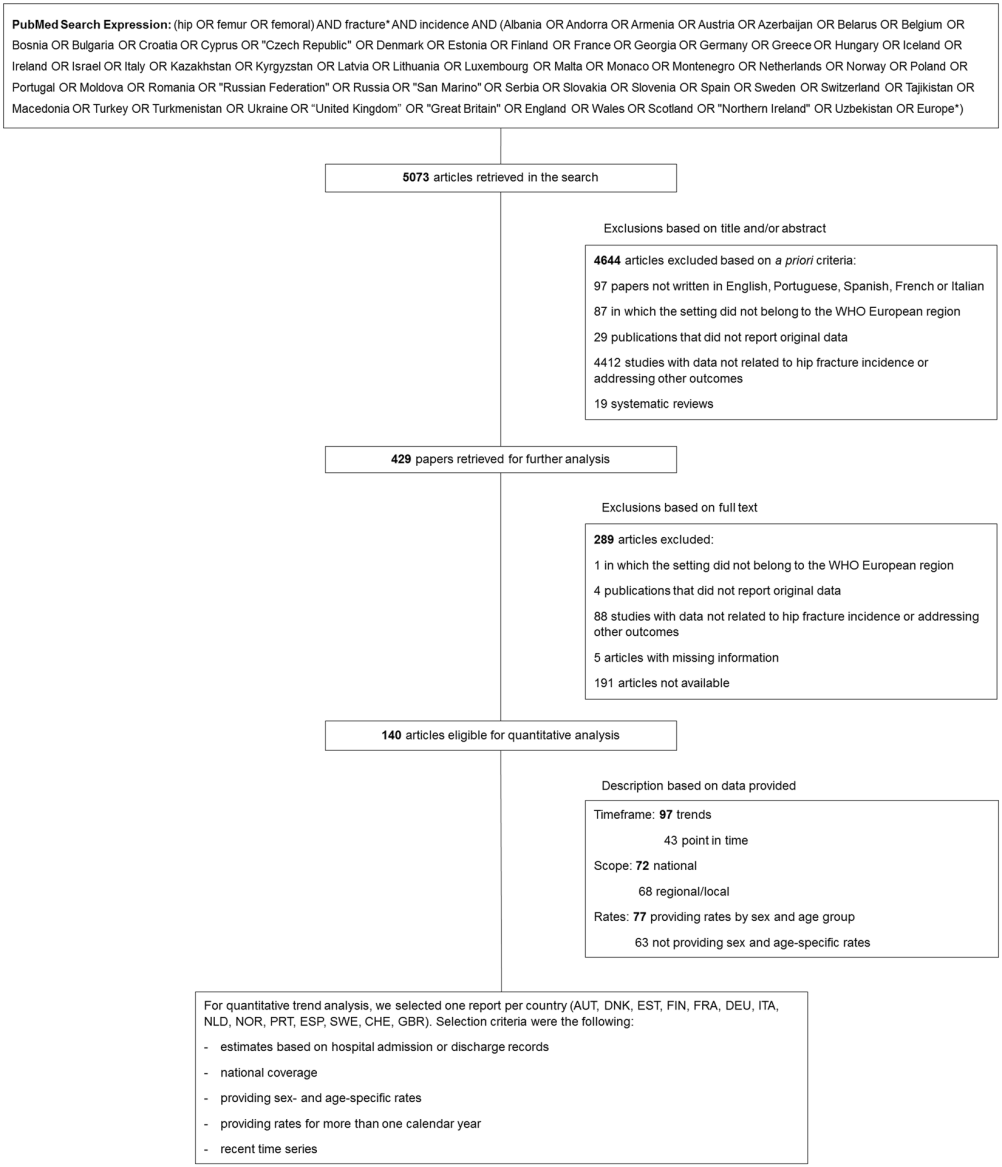
Flowchart of articles for data extraction. Abbreviations: WHO: World Health Organization, AUT: Austria, DNK: Denmark, EST: Estonia, FIN: Finland, FRA: France, DEU: Germany, ITA: Italy, NLD: the Netherlands, NOR: Norway, PRT: Portugal, ESP: Spain, SWE: Sweden, CHE: Switzerland, GBR: UK-England.

Studies were identified by searching Medline from inception to December 2015 for articles reporting trends of hip fracture incidence rates in European countries (Fig. 1). References were screened by two reviewers (AM, RL). In addition to the subject-matter filters detailed below, we applied the following formal exclusion criteria: i) articles not written in English, Portuguese, Spanish, French or Italian, ii) articles from populations outside the World Health Organization European Region, iii) non-eligible publication types (editorials, comments, guidelines, case reports and reviews), iv) studies that did not report incidence estimates, and v) studies that presented data for only one time point. Since we aimed to extract and model recent age- and sex-specific hip fracture incidence trends for different European countries, we defined methodological strategies to address the following specific issues:

- Validity of estimates: we selected only studies that calculated incidence rates by using as numerator the number of cases ascertained through hospital records in a defined time frame and as denominator the whole population in the catchment area, i.e. we excluded studies that presented incidence estimates obtained by following closed cohorts or through repeated surveys of hospitals;
- Replicability: we chose papers that provided sex- and age-specific incidence rates as part of their results or supplementary materials. In the case where estimates were provided in the graphical form, authors were contacted and asked to provide the raw incidence rates. All papers that did not present estimates that could be directly used as inputs for the model were excluded, even if they examined incidence trends. This included, for instance, papers that presented only age-standardized or model-predicted estimates;
- Geographic coverage: to improve comparability of target populations between countries, studies were eligible only if they covered the whole population of each country; those with regional coverage were excluded;
- Homogeneity: eligible papers used official hospital and demographic statistics to compute rates and that had national coverage. As such, it is very probable, and indeed desirable, that publications regarding the same country during the same time period have worked on the same raw data or its subsets. Further differences are likely due to specific methodological options such as diagnosis codes considered eligible, correction for readmissions or age-groups considered. Therefore, to avoid repetitions that would cause an artefactual increase in statistical power without adding information, we opted to use a single time series per country. The selection of the study to include was independent of the conclusion of that individual study with regard to the trend examined and was guided by model quality criteria defined on item e);
- Modelling quality: to improve model fit, when time series overlapped within the same country and year, we defined the following selection criteria, in order of preference: 1) wider time span, 2) more recent estimates and 3) shorter intervals between estimates. This implied that whenever there was more than one time series per country we started by excluding the series with the shortest time span. If time spans had the same width, we excluded the oldest series. In case calendar periods also overlapped, we excluded the one with the largest intervals between time points.

We identified eligible papers for the following 14 countries: Austria^9^, Denmark^17^, Estonia^24^, Finland^7^, France^25^, Germany^12^, Italy^26^, the Netherlands^27^, Norway^28^, Portugal^29^, Spain^30^, Sweden^31^, Switzerland^18^, and the UK – England^32^. Series differed between countries in time span, periodicity and age groups considered, but no data imputation was conducted (Table 1). From each paper, we extracted sex- and age-specific incidence rates of hip fracture for that country in each year. Estimates referred to the age groups defined in each paper and we indexed each rate to the midpoint of the corresponding age category.

**Table 1:** Characteristics of studies included.

Country	Author, publication year	Calendar period (no. of time points)	Age groups considered (years)	ICD revision and codes	Correction for multiple admissions
Austria	Dimai^9^	1989–2008 (20)	50–54, 55–59, 60–64, 65–69, 70–74, 75–79, 80–84, 85–89, 90–94, 95+	ICD-9: 820	Yes
				ICD-10: S72.0, S72.1, S72.2	
Denmark	Abrahamsen^17^	1997–2006 (10)	60–64, 65–69, 70–74, 75–79, 80–84, 85+	ICD-10: S72.0, S72.1	Yes
Estonia	Jürisson^24^	2005–2012 (8)	50–59, 60–69, 70–79, 80+	ICD-10: S72.0, S72.1, S72.2	Yes
Finland	Korhonen^7^	1970–2010 (41)	50–64, 65–74, 75–84, 85+	ICD-8 and ICD-9: 820	Yes
				ICD-10: S72	
France	Maravic^25^	2002–2008 (7)	40–59, 60–74, 75–84, 85+	ICD-10: S72.0, S72.1	No
Germany	Icks^12^	1995–2004 (10)	40–49, 50–59, 60–64, 65–69, 70–74, 75–79, 80–84, 85–89, 90+	ICD-9: 820	Yes
				ICD-10: S72.0, S72.1, S72.2	
Italy	Piscitelli^26^	2002–2008 (7)	40–44, 45–49, 50–54, 55–59, 60–64, 65–69, 70–74, 75–79, 80–84, 85–89, 90–94, 95+	ICD-9: 820.0-820.3, 820.9, 820.9, 821.1	Yes
Netherlands	Hartholt^27^	1991–2008 (5)	65–69, 70–74, 75–79, 80–84, 85–89, 90–94, 95+	ICD-9: 820	Yes
Norway	Omsland^28^	1999–2008 (10)	50–69, 70–74, 75–79, 80–84, 85–89, 90+	ICD-9: 820	Yes
				ICD-10: S72.0, S72.1, S72.2	
Portugal	Alves^29^	2000–2008 (9)	50–54, 55–59, 60–64, 65–69, 70–74, 75–79, 80–84, 85+	ICD-9: 820 and admission cause low to moderate trauma	No
Spain	Azagra^30^	1997–2010 (14)	65–69, 70–74, 75–79, 80–84, 85+	ICD-9: 820	No
Sweden	Nilson^31^	1987–2009 (23)	65–79, 80+	ICD-9: 820	Yes
				ICD-10: S72.0–S72.2	
Switzerland	Lippuner^18^	2000–2007 (8)	45–49, 50–54, 55–59, 60–64, 65–69, 70–74, 75–79, 80–84, 85+	ICD-10: S72.0, S72.1, S72.2	No
United Kingdom (England)	Wu^32^	1998–2008 (11)	45–54, 55–64, 65–74, 75–84, 85+	ICD-10: S72.0, S72.1, S72.2	Yes

### Modelling of the effects of age and calendar year

We started by building a model to describe the overall effects of calendar year and age using pooled data from all countries. Following our *a priori* hypothesis, the natural logarithms (log) of sex-specific fracture rates were modelled as a quadratic function of the calendar year. The well-known (fixed) effect of age was included as the fractional polynomial that best predicted the observed curvature. Both age and calendar year were standardized to zero mean and unit variance. The fixed-effects component of the models were of the form:

$$\begin{array}{rcl}\text{Women}:\,\mathrm{log}(\text{rate}) & \sim  & {{\rm{\beta }}}_{0}+{{\rm{\beta }}}_{1}{(\text{year}}^{\ast })+{{\rm{\beta }}}_{2}{{(\text{year}}^{\ast })}^{2}+{{\rm{\beta }}}_{3}{({{\rm{age}}}^{\ast }/100)}^{3}\\  &  & +{{\rm{\beta }}}_{4}{({{\rm{age}}}^{\ast }/100)}^{3}\,\mathrm{log}({{\rm{age}}}^{\ast }/100)+{{\rm{\varepsilon }}}_{{\rm{women}}}\\ \text{Men}:\,\mathrm{log}(\text{rate}) & \sim  & {{\rm{\beta }}}_{0}+{{\rm{\beta }}}_{1}{(\text{year}}^{\ast })+{{\rm{\beta }}}_{2}{{(\text{year}}^{\ast })}^{2}+{{\rm{\beta }}}_{3}{({{\rm{age}}}^{\ast }/100)}^{-2}\\  &  & +{{\rm{\beta }}}_{4}{({{\rm{age}}}^{\ast }/100)}^{-2}\,\mathrm{log}({{\rm{age}}}^{\ast }/100)+{{\rm{\varepsilon }}}_{{\rm{men}}}\end{array}$$

with residuals ε following a standard normal distribution. The existence of a significant variability at the country level among the rates was tested through the inclusion of random effects, fitted by restricted maximum likelihood^33^.

Random effects were considered: at the intercept level, to represent background fracture rates; on the linear effect of calendar year, to represent timing of trend reversal; and on the quadratic effect of calendar year, to represent tempo of trend reversal. For the evaluation of the goodness-of-fit and comparisons between models four indices were used: the Bayesian Information Criterion (BIC), the correlation coefficient between predicted and observed values, the Relative Squared Error (RSE) and the Relative Absolute Error (RAE). The RSE (resp. RAE) is defined as the ratio between the total squared (resp. absolute) error associated with the model prediction and the total squared (resp. absolute) error of the simple predictor of the average of the observed values^34^. The predicted values for the calendar year of trend reversal (h) and the maximum incidence rate (k), for the sample mean age, were computed as the coordinates of the vertex of each country-specific parabola:

$${\rm{h}}=-\,{{\rm{\beta }}}_{1}/(2\cdot {{\rm{\beta }}}_{2}),\,{\rm{k}}=\exp [(4\cdot {{\rm{\beta }}}_{2}\cdot {{\rm{\beta }}}_{0}-{{\rm{\beta }}}_{1}^{2})/(4\cdot {{\rm{\beta }}}_{2})]$$

### Model-based country clustering

Once the models were obtained, the country specific features exhibited by the models random coefficients were clustered by Gaussian mixture models^35^. The aim was to group countries according to their fitted features over time. Gaussian mixture modelling is a probabilistic method to identify homogeneous subgroups within a population assuming that the observed data come from a mixture of a finite number of Gaussian distributions. The characteristics (orientation, volume and shape) of the distributions are estimated from the data and can be allowed to vary between clusters or constrained to be the same for all clusters^36^. For each sex, we selected a cluster solution from among the three with the lowest BIC (Supplementary material)^37^. The interpretation of the clusters was based on the values of the peak incidence rates and timing of trend reversal.

Data analysis was carried out with the software R 2.14.1^38^ using the packages mfp^39^, nlme^40^, and mclust^41^ for model fitting by fractional polynomials, mixed-modelling and model-based clustering, respectively.

## Results

**Figure 2:**
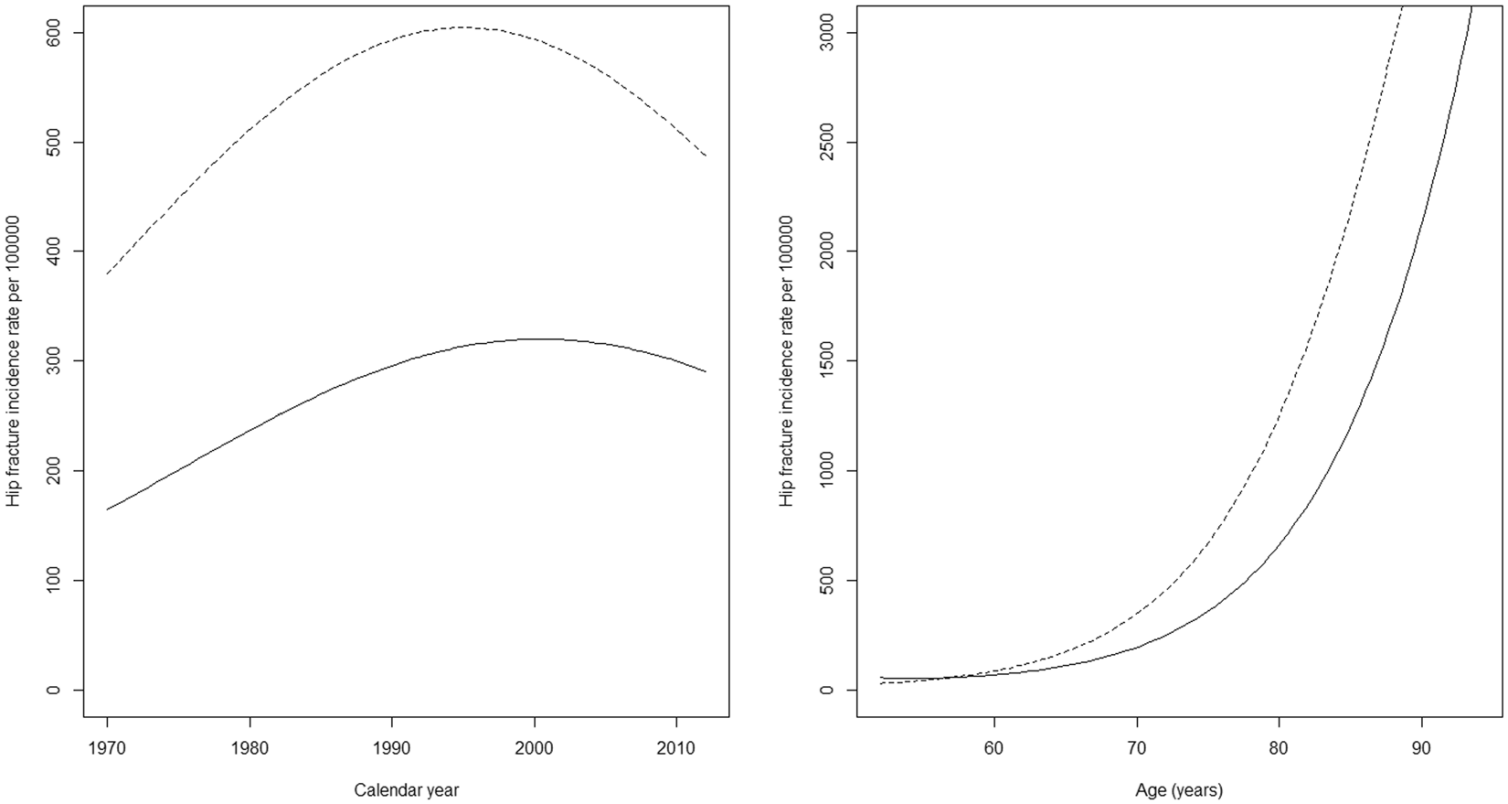
Predicted fixed effects of calendar year and age on hip fracture incidence in women (dashed line) and men (solid line). To plot predicted values, the effect of calendar year was modelled for the average age in the sample (74 years) and the effect of age modelled for the average calendar year (2000).

Figure 2 shows the predicted average trajectories of hip fracture incidence rates over calendar year and age. From the fixed effects model component, the predicted year of trend reversal was on average 1999 in women (at a peak incidence rate of about 600 per 100 000) and 2000 in men (about 300 per 100 000 men). Plots of age effects (Fig. 2) confirmed the dramatic increase in incidence rates with ageing, occurring at a faster rate in women. Observed and predicted fracture rates in each country are shown as Supplementary material.

When modelling country-level random effects (Table 2), we found that including random terms for the intercept and year slope improved the variability explained: compared to a model with fixed effects only, Model 1 (random intercept) explained 47.6% of the residual variability in women and 56.6% in men, whereas in Model 2 (random intercept and year slope), these proportions were 48.6% and 57.4%, respectively. Improvements from Model 1 to 2 were significant, as assessed through the likelihood ratio test. Adding a random quadratic effect of year did not improve the model’s predictive ability. Goodness-of-fit indices also suggested good adjustment of Model 2 to the observed data, with no substantial improvement in Model 3. In practical terms, this indicates that countries differed regarding background fracture rates and timing of trend reversal, whereas the tempo of trend reversal did not show significant intercountry variability.

**Table 2:** Summary of the adjustment of mixed effects models, including random effects for country, to observed hip fracture incidence rates.

Random effects	Model 1		Model 2		Model 3	
	Women	Men	Women	Men	Women	Men
SD_intercept_	0.348	0.454	0.348	0.457	0.346	0.457
SD_year(linear)_	—	—	0.060	0.077	0.060	0.077
SD_year(quadratic)_	—	—	—	—	0.014	0.000
SD_residual_	0.176	0.189	0.173	0.185	0.173	0.185
p-value (LR test, each model vs. the previous)	—	—	<0.001	<0.001	0.860	>0.999
Proportion (%) of variability explained compared to the fixed effects only model	47.6	56.6	48.6	57.4	48.5	57.4
Bayesian Information Criterion (BIC)	−506.3618	−352.0886	−517.2905	−364.5618	−497.2848	−343.7998
Correlation between predicted and observed values	0.9935	0.9906	0.9938	0.9910	0.9938	0.9910
Relative Squared Error (RSE)	1.281	1.864	1.230	1.778	1.232	1.778
Relative Absolute Error (RAE)	9.997	12.193	9.784	11.935	9.781	11.935

SD - standard deviation; LR - likelihood ratio.

Model 1: random intercept.

Model 2: Model 1 + random linear term for calendar year.

Model 3: Model 2 + random quadratic term for calendar year.

Table 3 presents the predicted peak hip fracture incidence rates and calendar years of trend reversal by country, including extrapolated estimates for countries where the reversal was predicted outside of the time span covered by empirical data. Predicted peak fracture rates were lowest in Portugal (376.0 per 100 000 women and 156.9 per 100 000 men) and highest in Sweden (1389.8 per 100 000 women and 742.4 per 100 000 men). In terms of timing, the earliest trend reversals were predicted between 1986 and 1987 among women in Sweden and Denmark, whereas in men the earliest reversals were predicted between 1990 and 1992 in Switzerland, Denmark and Sweden.

**Table 3:** Predicted values for maximum hip fracture incidence rate and calendar year of trend reversal using a mixed effects model.

Country	Predicted maximum hip fracture incidence rate (per 100 000)		Predicted calendar year of trend reversal		Time span of empirical data	Age range (years)
	Women	Men	Women	Men		
Austria	677.8	389.3	2001.0	2008.4	1989–2008	50–95+
Denmark*	1089.7	551.1	1987.1	1991.9	1997–2006	60–85+
Estonia*	531.0	468.7	1995.1	1999.2	2005–2012	50–80+
Finland	649.5	429.8	1995.2	2002.6	1970–2010	50–85+
France*	461.9	217.6	1994.9	2000.5	2002–2008	40–85+
Germany	527.4	279.5	1997.0	2004.8	1995–2004	40–90+
Italy*	526.5	237.5	1997.7	2002.2	2002–2008	40–95+
Netherlands	516.1	283.5	1994.8	1999.8	1991–2008	65–95+
Norway*	969.1	554.4	1993.0	1998.2	1999–2008	50–90+
Portugal*	376.0	156.9	1993.9	2000.6	2000–2008	50–85+
Spain	420.0	195.0	1996.8	2004.0	1997–2010	65–85+
Sweden	1389.8	742.4	1986.8	1992.4	1987–2009	65–80+
Switzerland*	647.0	335.4	1991.6	1990.1	2000–2007	45–85+
United Kingdom (England)	472.1	214.9	1998.9	2007.8	1998–2008	45–85+

*Countries where model-predicted estimates of reversal year and peak rates were extrapolated (outside the time span covered by empirical data).

**Figure 3:**
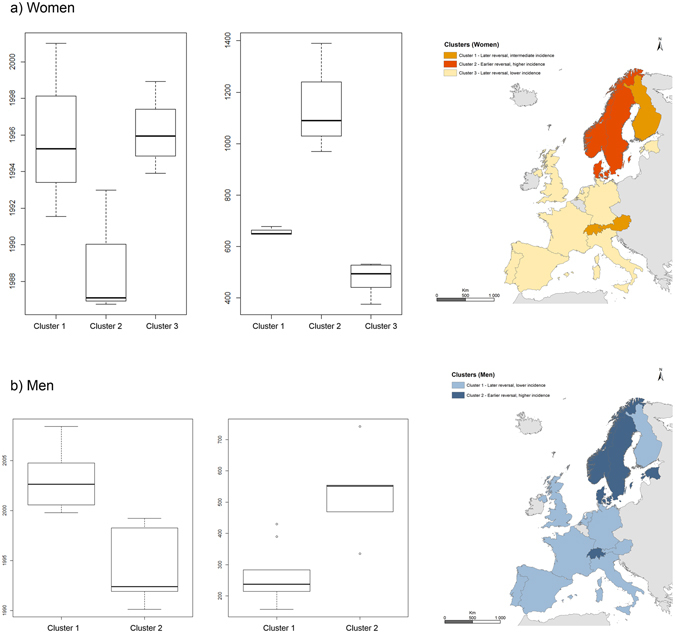
Distribution of calendar year of trend reversal (boxplots on the left) and peak incidence rates (boxplots on the right) in country clusters and the geographical distribution of countries in each cluster (panel a: women; panel b: men). Maps generated with ArcGIS version 10.3, by the Environmental Systems Research Institute (ESRI).

After extracting country-level random coefficients and applying mixture models, we found appropriate cluster solutions with three country groups for women and two groups for men. The distributions of coefficients for background rates (k) and timing of reversal (h) are presented in Fig. 3, together with maps showing the geographical distribution of country clusters.

In women, clusters were characterized by:

1. A later trend reversal and an intermediate peak incidence: Austria, Finland, and Switzerland, where fractures peaked on average in the mid-1990s with incidence rates between 600 and 700 per 100 000 women.
2. An earlier trend reversal and a higher peak incidence: Denmark, Norway and Sweden, where fracture rates peaked on average in the late 1980s with average incidence rates above 1000 per 100 000 women.
3. A later trend reversal and a lower peak incidence: Estonia, France, Germany, Italy, the Netherlands, Portugal, Spain, and the UK (England), where rates peaked on average in the mid-1990s with levels below 600 per 100 000 women.

Among men, clusters were:

1. A later trend reversal and a lower peak incidence: Austria, Finland, France, Germany, Italy, the Netherlands, Portugal, Spain, and the UK (England), where fractures peaked on average in the early 2000s with an average incidence below 300 per 100 000 men.
2. An earlier trend reversal and a higher peak incidence: Denmark, Estonia, Norway, Sweden, and Switzerland, where fracture rates peaked on average in the mid-1990s with an average incidence above 500 per 100 000 men.

## Discussion

We found that recent time trends of hip fracture incidence in 14 European countries can be described within the same overall pattern of secular trend reversal: rates have evolved approximately as a quadratic function of time with significant intercountry variability in the timing of reversal and peak fracture rates.

Previous evidence syntheses involving different countries examined specific aspects of the hip fracture epidemic within each country using aggregate measures such as annual percent change^21, 22^. These approaches are quite useful to examine break points, but are mostly interpretable at the country level and do not allow for quantitatively assessing whether the trend is generalizable to different settings. In the present work, we extracted sex- and age-specific rates for each country, and tested an overall quantitative behaviour, while allowing for country-level variability. To the best of our knowledge, this is the first study to bring together two key features of hip fracture epidemiology: background fracture rates and timing of trend reversal. Another important contribution was the identification of clusters from model-based statistical similarities rather than by imposing geographical proximity.

Our main modelling assumption was that trends could be described as a quadratic function of time in all countries. This option allowed to estimate a reversal timing for each country even when country-specific empirical data did not capture an equivalent time frame, in terms of epidemiological meaning, in all settings. However, oscillations that would produce the same or different adjustment to the model may have occurred outside of the time span considered or between two time points modelled. As such, reversal timing predicted at the country-level – which, as described in Table 3, was extrapolated beyond the time span covered by observed data for a number of countries – should be interpreted with caution and bearing in mind the *a priori* assumption of a single shape for all countries. Nevertheless, the option for a quadratic function seems plausible both mechanistically and empirically. As a multifactorial outcome resulting from multiple influences throughout life^42^, we expect the risk of fragility fracture to show smooth rather than abrupt changes over time. Empirically, our assumption is supported both by the fit indices of our mixed-effects model and by the fact that most papers included in our analysis either focus on describing trend reversals or show data where a previous or concurrent reversal is plausible even if not specifically tested. A further validation is the external replication of reversals in studies not included, e.g. outside of the European region^43, 44^ or using alternative methodological approaches^45^. Regarding the effect of age, we opted to use categories as defined in each publication and their midpoint as the index age. This was based on an assumption of constant risk within each age group, which might be unrealistic, especially when classes are wide. However, most countries used 5-year age groups, where such an assumption is more likely to hold. In addition, our estimates of age effect on fracture incidence seem consistent with a wealth of previous knowledge^21, 46^.

We found that the behaviour of hip fracture incidence over time could be explained as a function of timing and background rates and that the tempo of reversal was not a key source of variability. It should be noted that country-level effects represent variability not only of background fracture rates and timing of trend reversal – which were our main focus – but also of methodological options in each paper whose data we used, since estimates may not be homogeneous between countries regarding coverage, case definition or additional eligibility criteria. However, hip fractures in high-income countries are rarely managed without hospital admission, which is in favour of comparable coverage between countries. Regarding case definition, all papers adopted ICD coding, but both the 9th and 10th revisions were used and not all eligible codes overlapped between studies. In addition, some estimates were corrected for multiple admissions, while others were not. Despite that, we have no reason to suspect substantial variations in the validity of estimates over time within each country. Since our main goal was to capture the overall shape of the trend in each country, rather than to produce country comparisons at each time point, we do not believe that these issues have seriously affected our main findings. Indeed, our cluster grouping of countries is largely compatible with previous stratification on the basis of background rates^47^, especially regarding Scandinavia and Southern Europe, which supports that our model does distinguish the relative burden between settings. A particularly clear example reflected in our cluster solution is that of Estonia, where men have incidence rates close to those of Scandinavia, while women show rates similar to those observed in Southern Europe^24^. A specific case where the present model was not consistent with long-term historical data was the UK. Indeed, fracture rates were reported to have levelled off before 1985, with a breakpoint in 1978^5^. We obtained a predicted peak rate at a much later date, which results from our choice of a later time series where such a breakpoint would, by definition, not be captured^32^. An interpretation may be that, up to the 1980s, the UK had an epidemic closer to that observed in Scandinavia, which later stabilized at lower levels, producing a recent behaviour more similar to that observed in Central and Southern Europe, with a second, smoother breakpoint captured by our cluster assignment. An interesting explanation for the cluster structure we propose (where there is statistical dependence between peak rates and timing of reversal) might be a lower threshold for trend reversal in countries with higher incidence. It seems indeed plausible that the fraction of the population in whom a fracture event was prevented was probably at a lower risk to begin with and was therefore more likely to have benefited from an overall decreasing trend.

Several mechanisms have been proposed to explain the widespread improvement in fracture rates^22^. A set of hypotheses relates to planned efforts targeting bone fragility, such as increasing availability of bone density testing, a growing uptake of pharmacological treatment of osteoporosis and menopausal hormone therapy, or better clinical management after a first fracture. However, trend reversals in many countries began before these strategies were widespread^5^. Additionally, reversals have been described in both sexes whereas osteoporosis management has much higher coverage (even though with a low population impact) in women^17^. Another set of hypotheses relates to macro-level changes that may have caused decreasing fracture trends as an unpredicted effect. Improvements in health and nutrition throughout the 20th century likely increased adult peak bone mass in younger cohorts, which is an early determinant of fracture^48^. As for shorter term risk factors, prosperity also brought about an increased frequency of overweight which, despite its deleterious effects, is protective of fragility fractures in adults. Additional important changes may include increased physical activity, and less frequent smoking and drinking habits^28^. There is also some empirical evidence that upstream factors that may influence trauma severity, such as urbanization, have followed similar time trends as hip fractures^22^. It should be noted, however, that our study did not aim to disentangle short- from long-term effects on trends, such as those that would be obtained from a formal age-period-cohort analysis. Thus, our findings regarding the effects of calendar period and age describe the hip fracture epidemic at the turn of the century but partly reflect earlier birth cohort influences, which amount to periods long before the fracture event itself and result from accumulated exposures throughout the life course.

Even though several explanations for trend reversals have been put forward, assessing their plausibility is challenging. Specifically, our findings suggest that the determinants of hip fracture trends may have evolved in different ways between high- and low-risk countries. Our proposal of a single shape to model secular trends in different countries provides a basis for examining candidate explanations in several settings simultaneously.

This study shows that it is plausible that recent hip fracture incidence rates in European countries can be described within the same overall pattern of secular trend reversal, with significant intercountry variability in the timing of reversal and peak fracture rates. This provides a quantitative basis for mechanistic explanations and may ultimately contribute to policy modelling.

## Electronic supplementary material

Supplementary materials
