# Supplementary material for: Quantitative modelling of hip fracture trends in 14 European countries: testing variations of a shared reversal over time
Source: Sci Rep. 2017 Jun 16;7:3754. doi: 10.1038/s41598-017-03847-x (PMC5473829; doi:10.1038/s41598-017-03847-x)

SUPPLEMENTARY MATERIALS

Manuscript title: Quantitative modelling of hip fracture trends in 14 European countries: testing variations of a shared reversal over time

Authors: Raquel Lucas[1,2], PhD; Ana Martins[1,2], MSc; Milton Severo[1,2], PhD; Poliana Silva[1], MSc; Teresa Monjardino[1,2], MSc, Ana Rita Gaio[3], PhD, Cyrus Cooper[4,5], PhD, FMedSci; Henrique Barros[1,2], MD PhD

Affiliations:

1. EPIUnit – Institute of Public Health, University of Porto, Porto, Portugal

2. Department of Clinical Epidemiology, Predictive Medicine and Public Health, University of Porto Medical School, Porto, Portugal

3. Department of Mathematics, Faculty of Sciences, University of Porto, Porto, Portugal

4. MRC Lifecourse Epidemiology Unit, University of Southampton, Southampton, UK

5. Southampton General Hospital, Southampton, UK

Corresponding author:

Raquel Lucas
Institute of Public Health, University of Porto
Rua das Taipas, 135
4050 600 Porto, Portugal
Email: rlucas@med.up.pt
Office phone: +351222061820; Mobile: +351962860846

Supplementary figures 1 to 28: Observed and predicted hip fracture incidence rates by age group, sex and country.

Supplementary figures 29 and 30: Bayesian information criteria for different cluster solutions in women and men.

Supplementary figure 1. Observed (red) and predicted (blue) hip fracture incidence rates by age group (from bottom left to top right), Austria - women

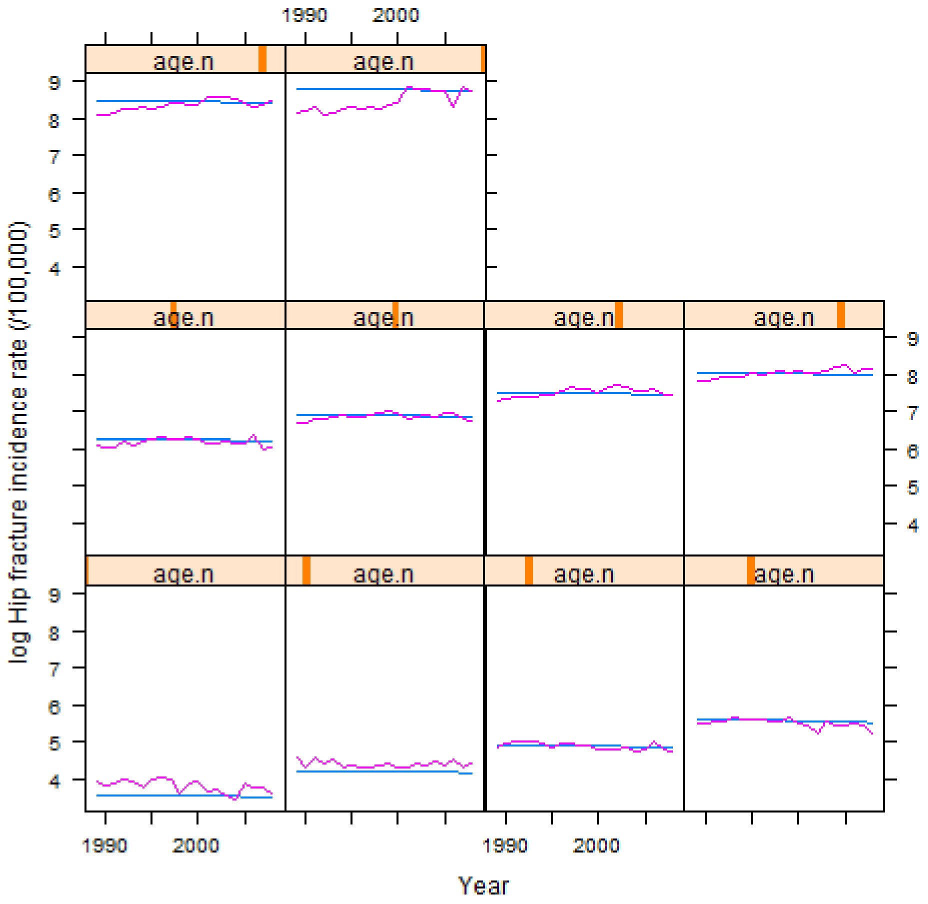

Supplementary figure 2. Observed (red) and predicted (blue) hip fracture incidence rates by age group (from bottom left to top right), Austria – men

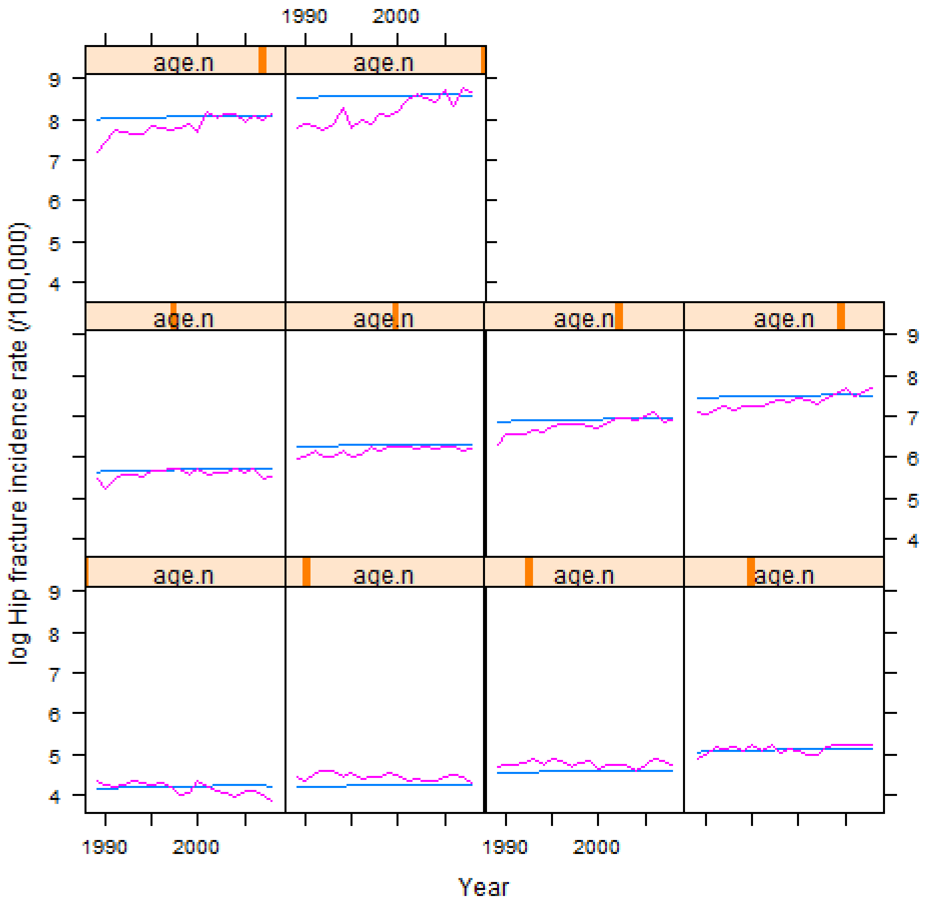

Supplementary figure 3. Observed (red) and predicted (blue) hip fracture incidence rates by age group (from bottom left to top right), Denmark – women

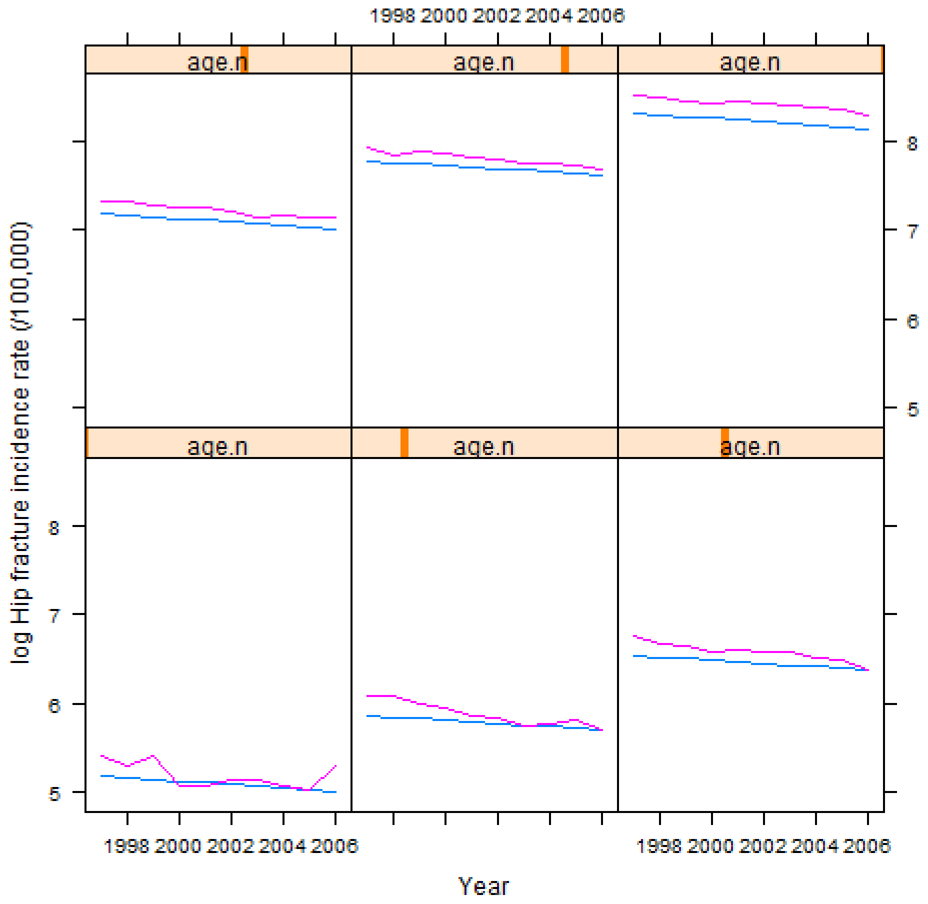

Supplementary figure 4. Observed (red) and predicted (blue) hip fracture incidence rates by age group (from bottom left to top right), Denmark – men

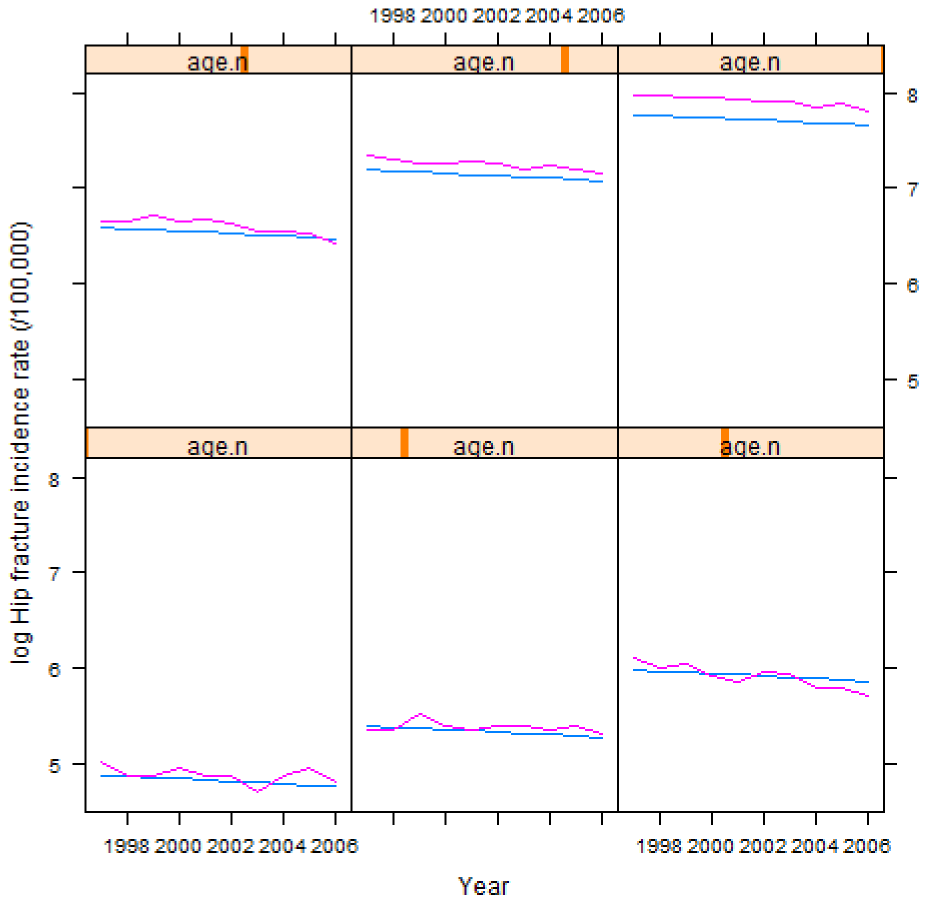

Supplementary figure 5. Observed (red) and predicted (blue) hip fracture incidence rates by age group (from bottom left to top right), Estonia – women

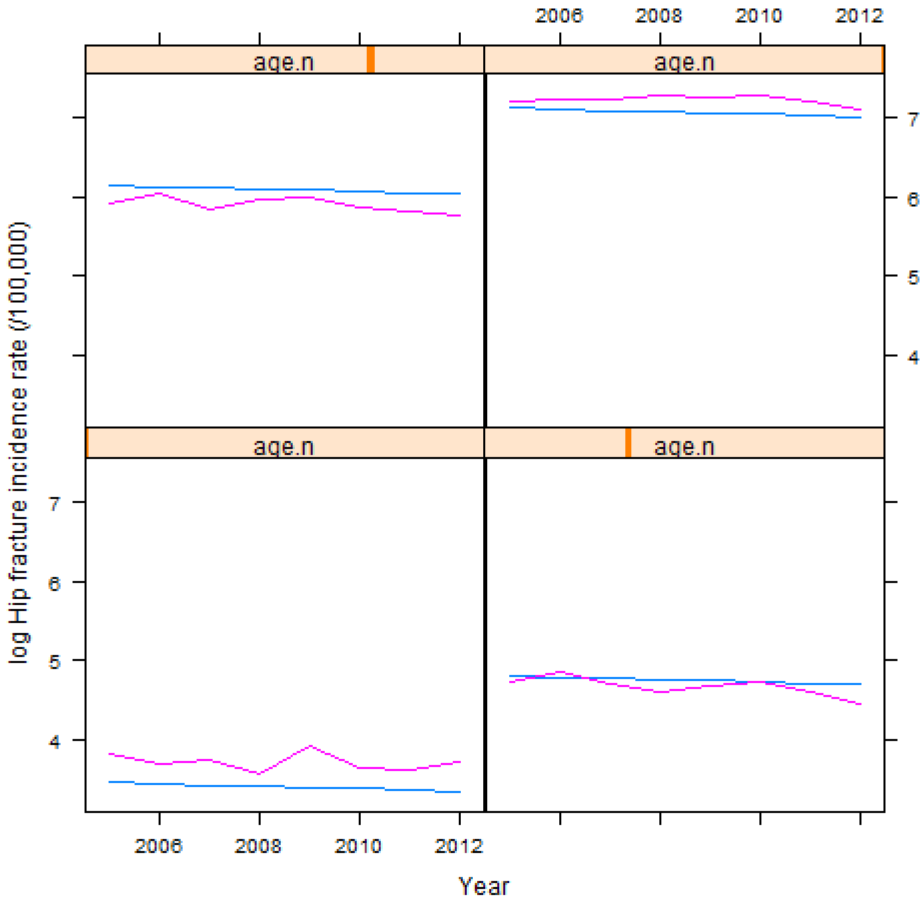

Supplementary figure 6. Observed (red) and predicted (blue) hip fracture incidence rates by age group (from bottom left to top right), Estonia – men

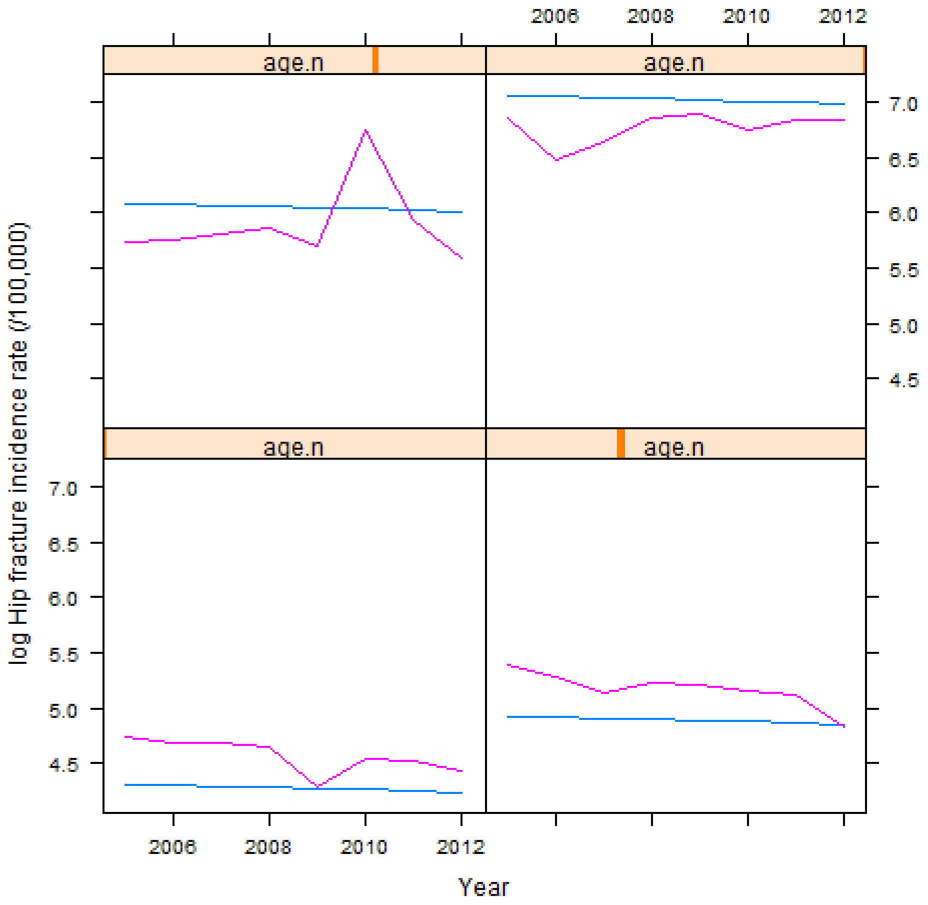

Supplementary figure 7. Observed (red) and predicted (blue) hip fracture incidence rates by age group (from bottom left to top right), Finland – women

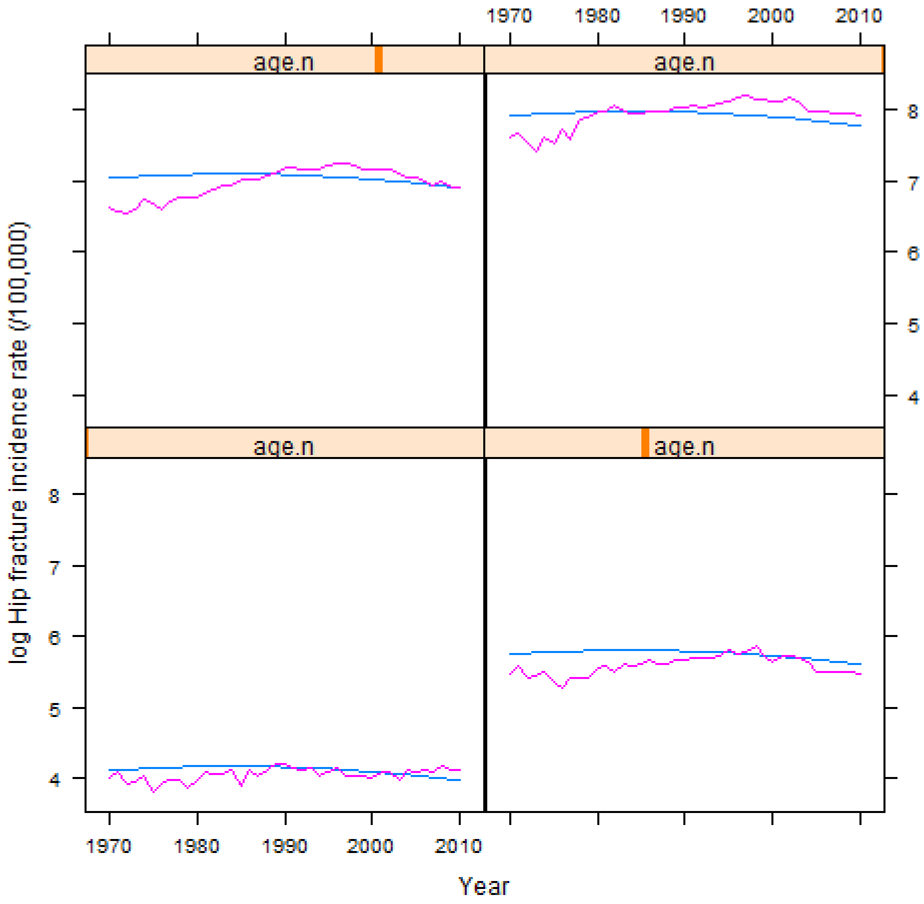

Supplementary figure 8. Observed (red) and predicted (blue) hip fracture incidence rates by age group (from bottom left to top right), Finland – men

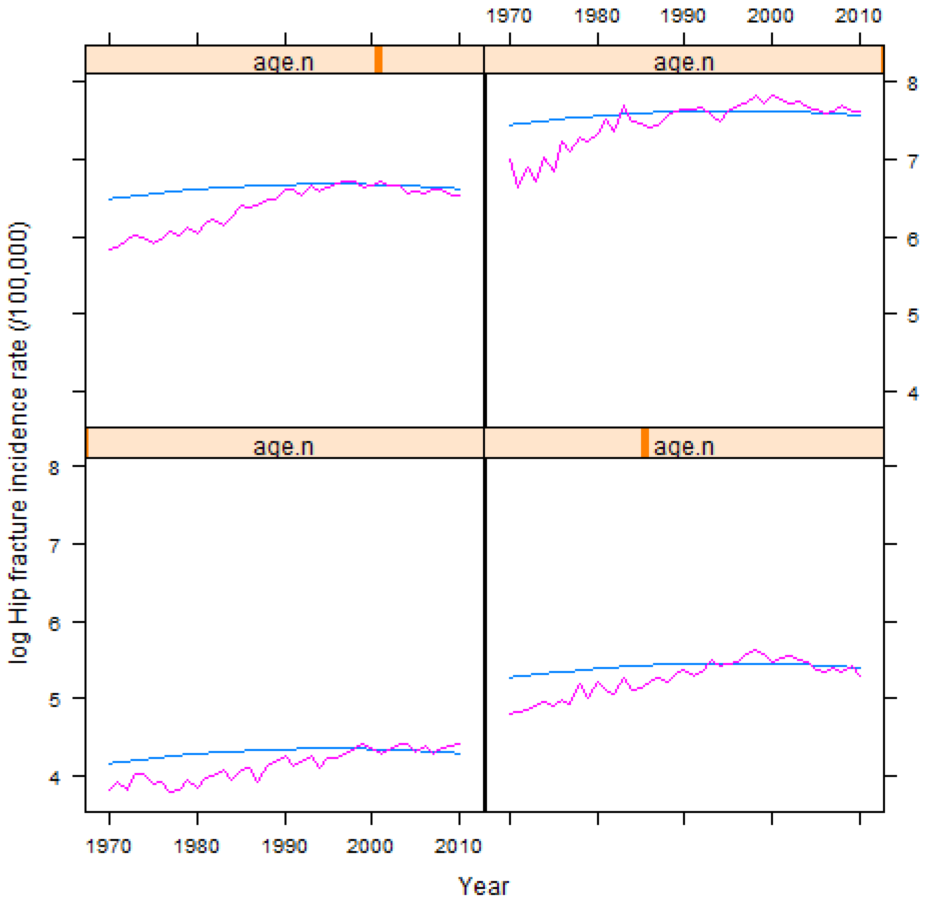

Supplementary figure 9. Observed (red) and predicted (blue) hip fracture incidence rates by age group (from bottom left to top right), France – women

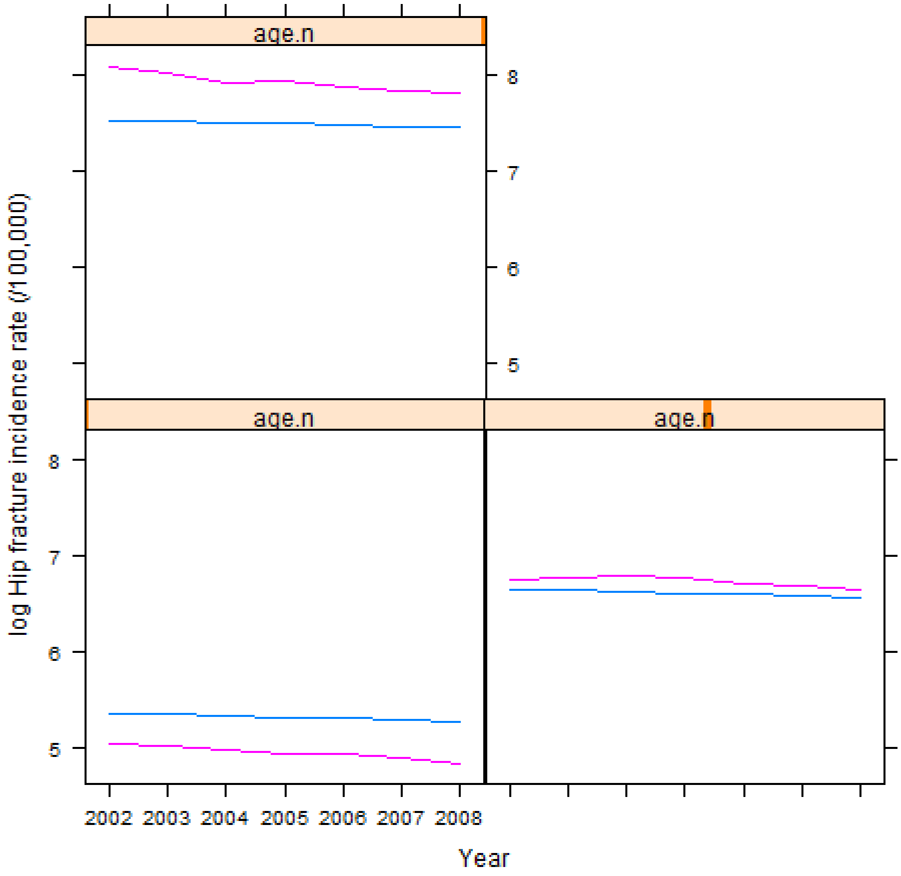

Supplementary figure 10. Observed (red) and predicted (blue) hip fracture incidence rates by age group (from bottom left to top right), France – men

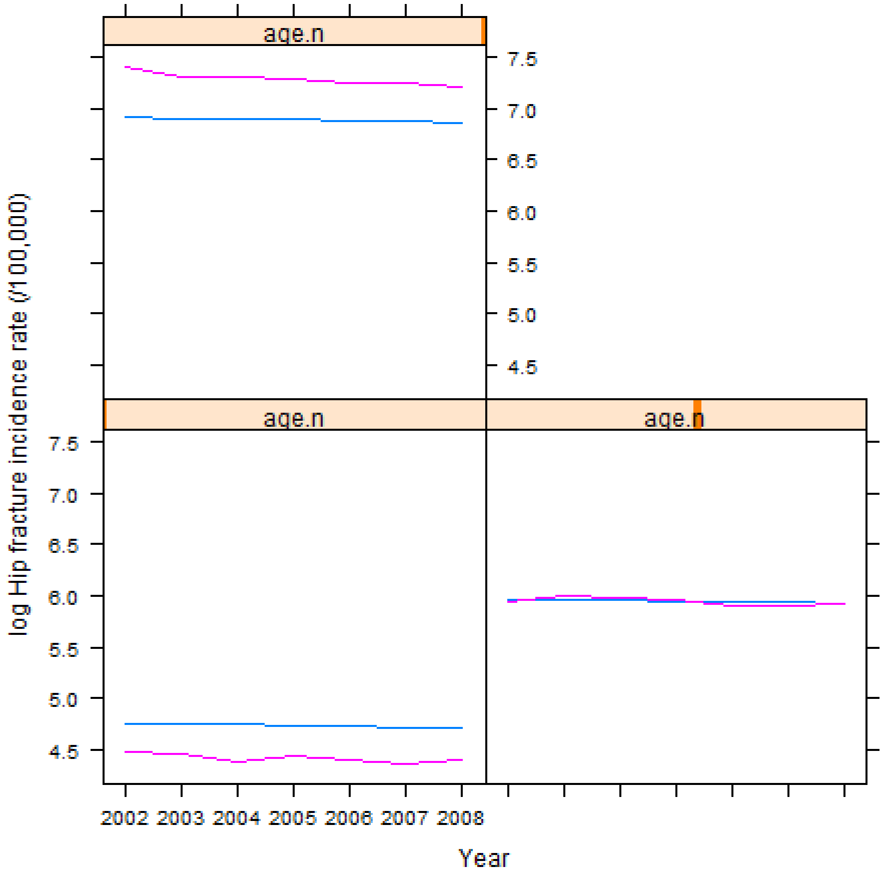

Supplementary figure 11. Observed (red) and predicted (blue) hip fracture incidence rates by age group (from bottom left to top right), Germany – women

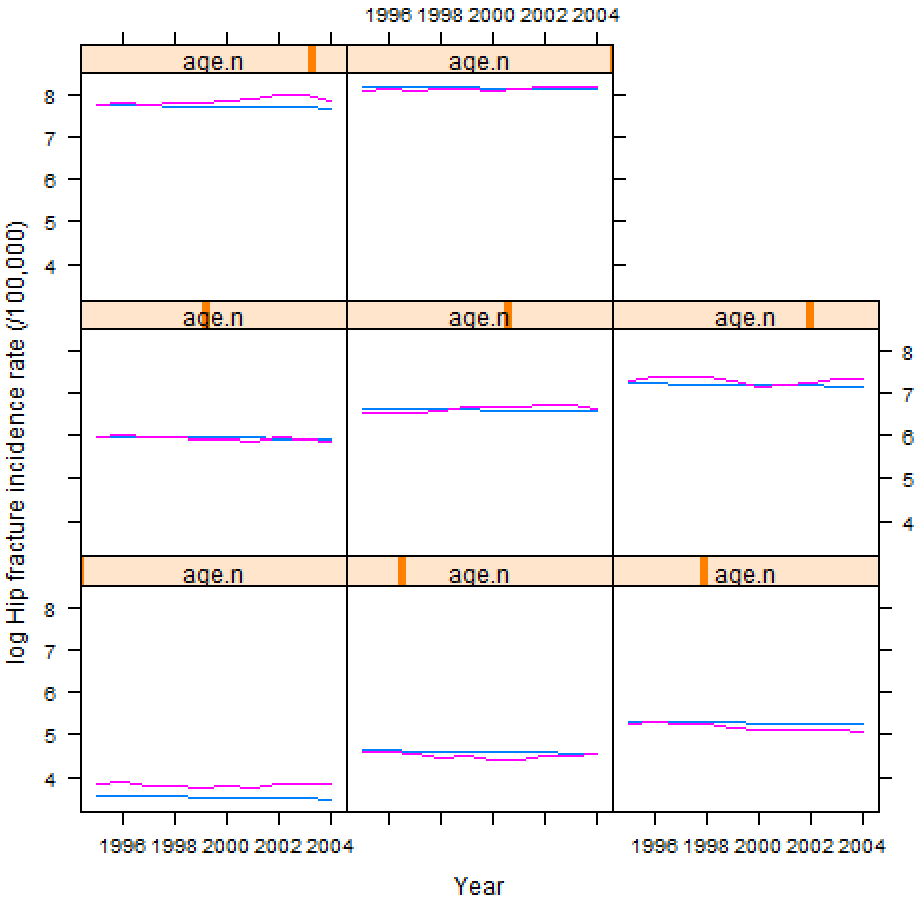

Supplementary figure 12. Observed (red) and predicted (blue) hip fracture incidence rates by age group (from bottom left to top right), Germany – men

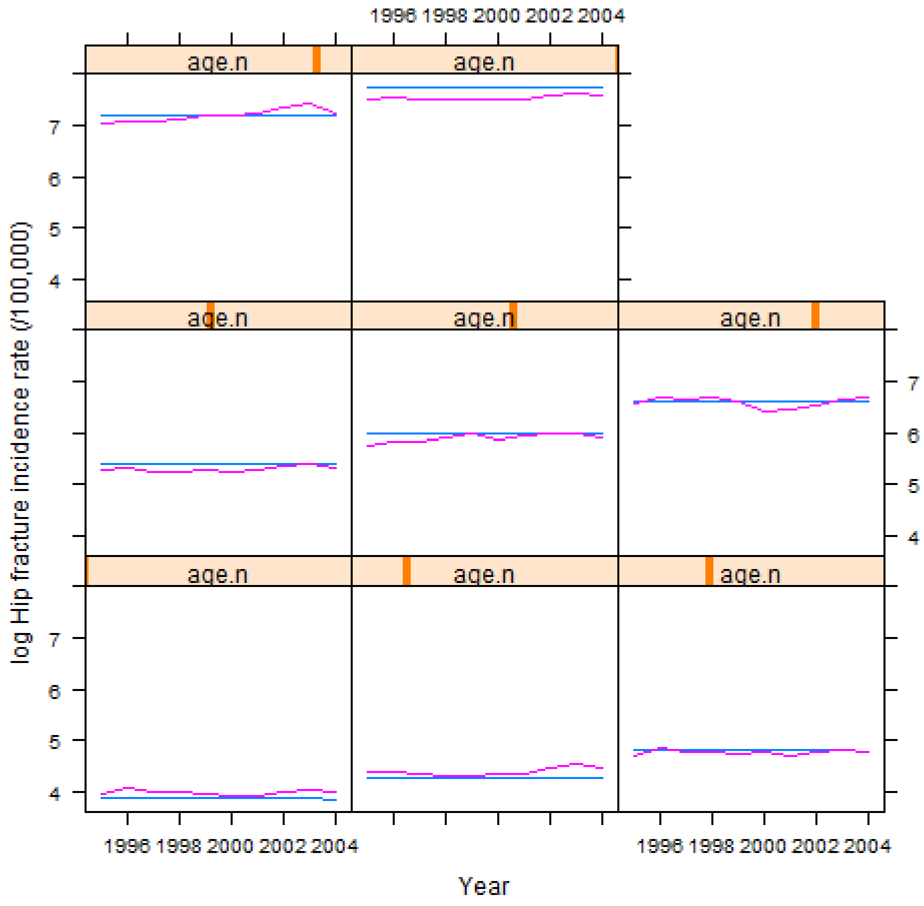

Supplementary figure 13. Observed (red) and predicted (blue) hip fracture incidence rates by age group (from bottom left to top right), Italy – women

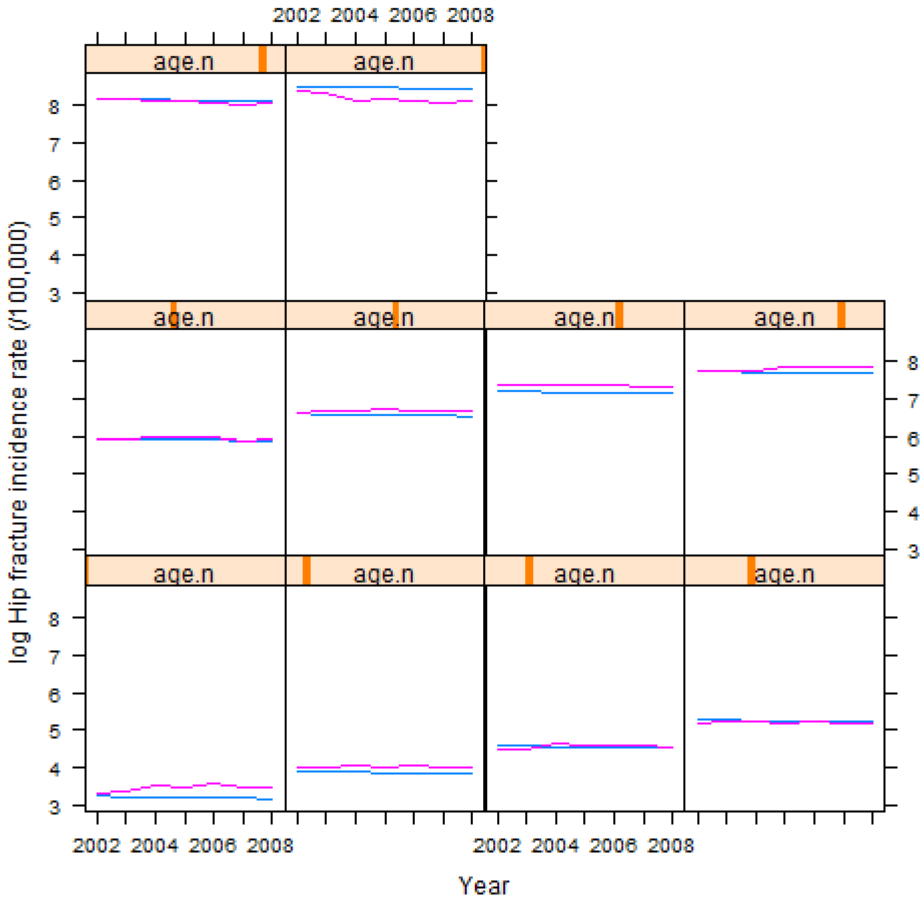

Supplementary figure 14. Observed (red) and predicted (blue) hip fracture incidence rates by age group (from bottom left to top right), Italy – men

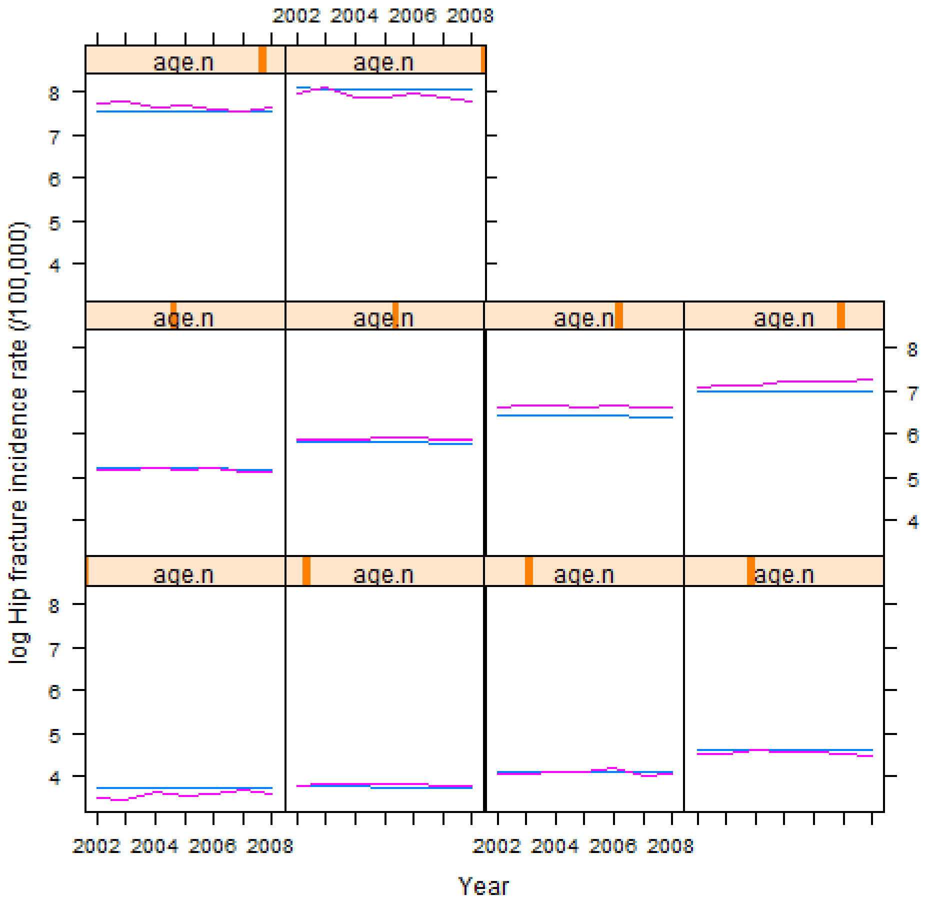

Supplementary figure 15. Observed (red) and predicted (blue) hip fracture incidence rates by age group (from bottom left to top right), Netherlands – women

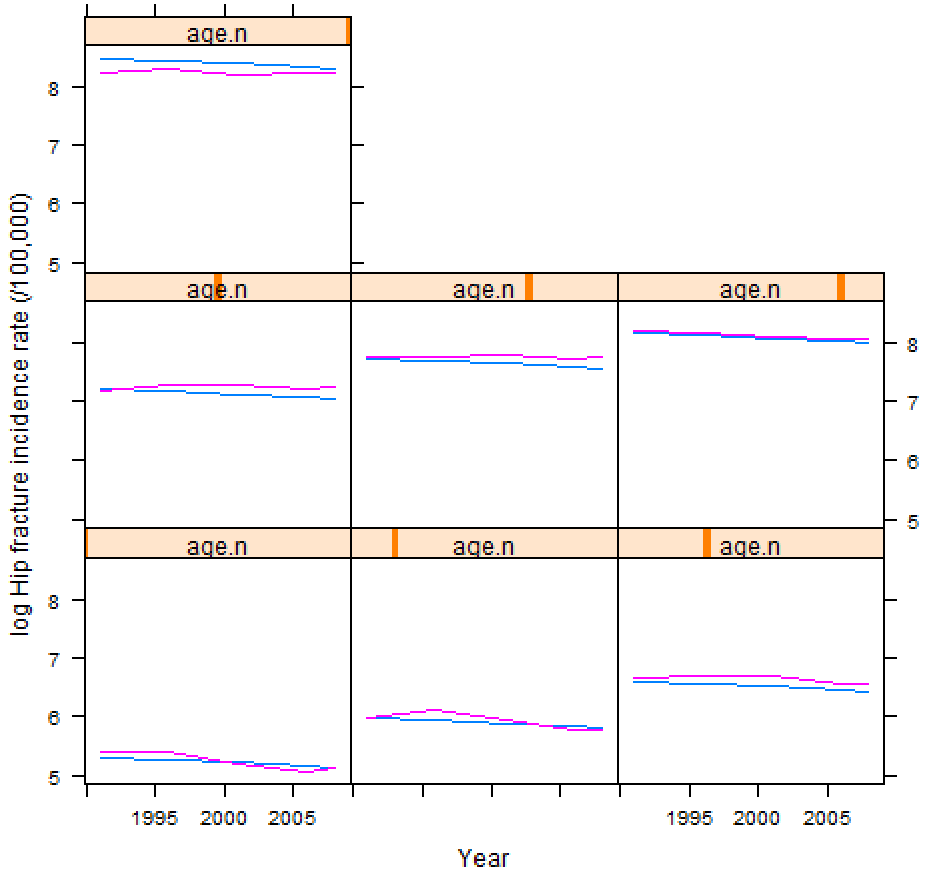

Supplementary figure 16. Observed (red) and predicted (blue) hip fracture incidence rates by age group (from bottom left to top right), Netherlands – men

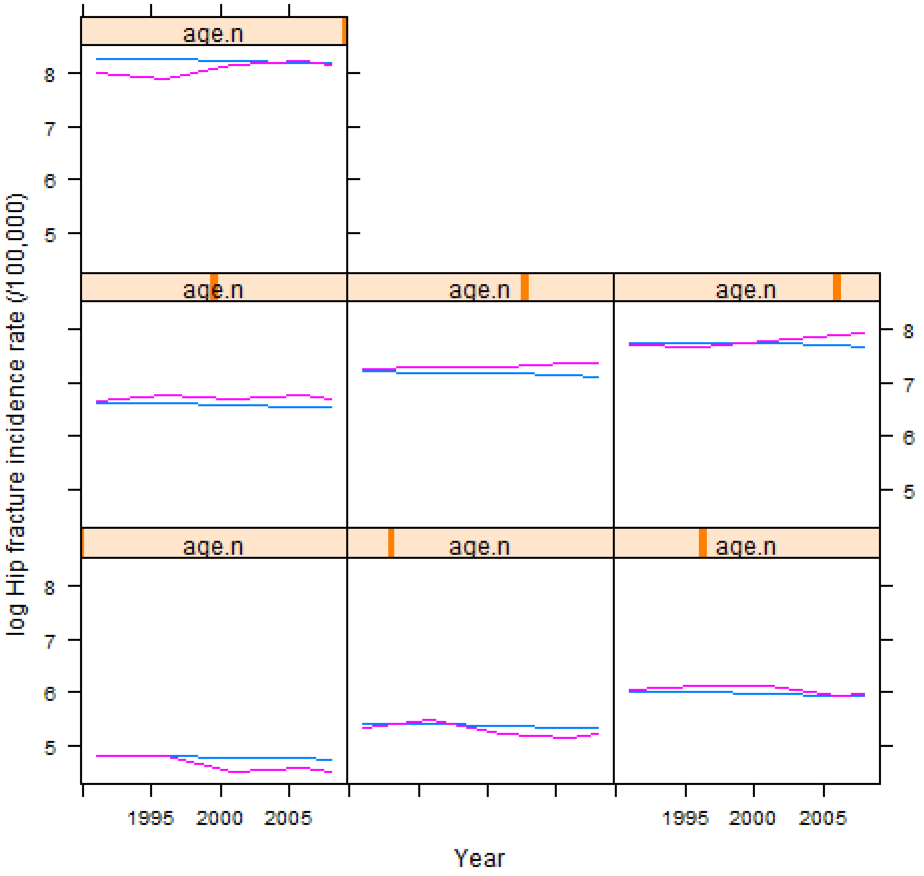

Supplementary figure 17. Observed (red) and predicted (blue) hip fracture incidence rates by age group (from bottom left to top right), Norway – women

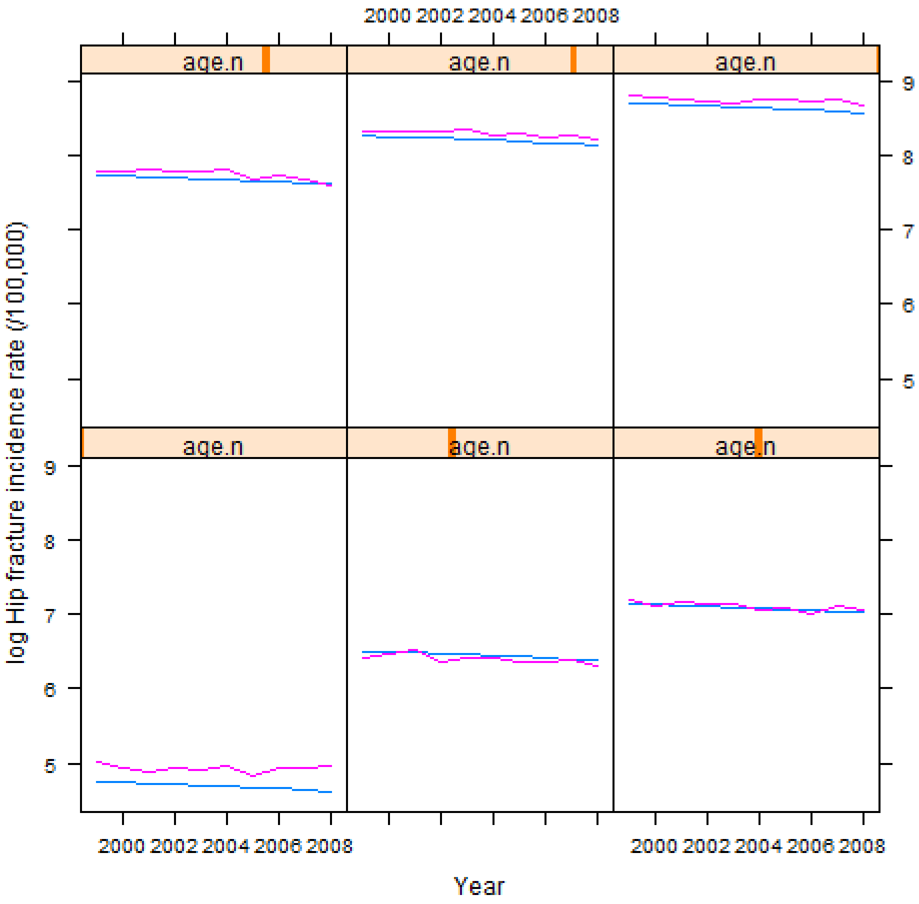

Supplementary figure 18. Observed (red) and predicted (blue) hip fracture incidence rates by age group (from bottom left to top right), Norway – men

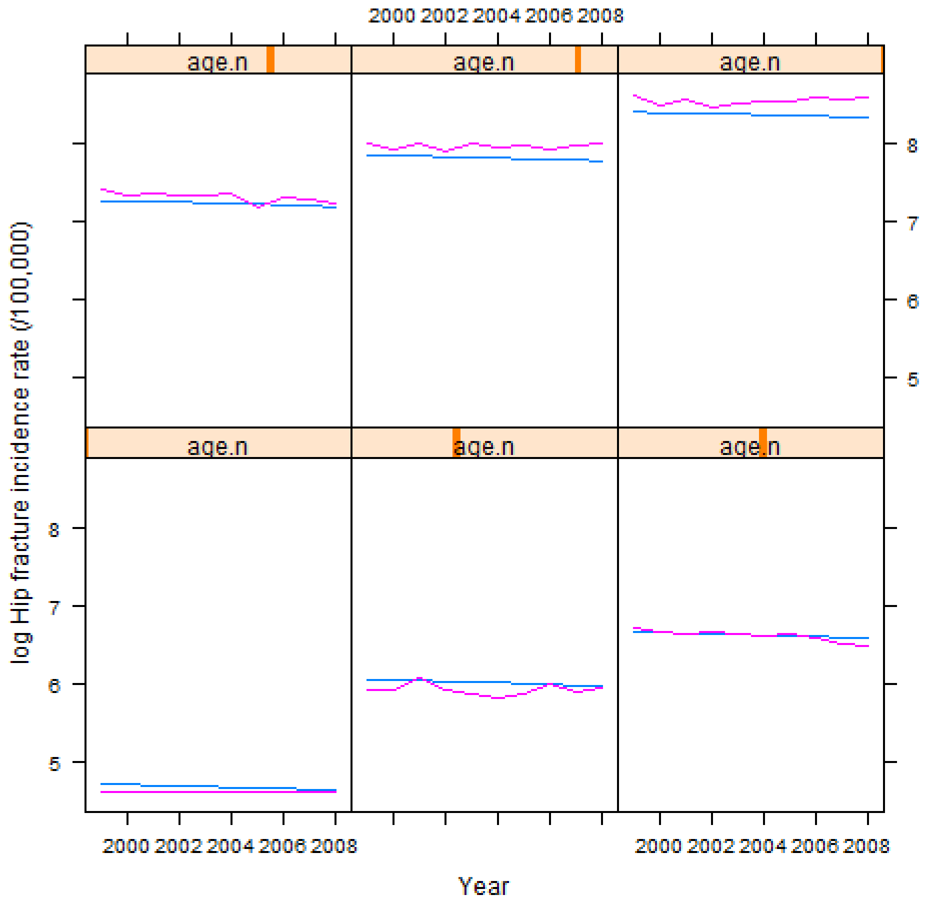

Supplementary figure 19. Observed (red) and predicted (blue) hip fracture incidence rates by age group (from bottom left to top right), Portugal – women

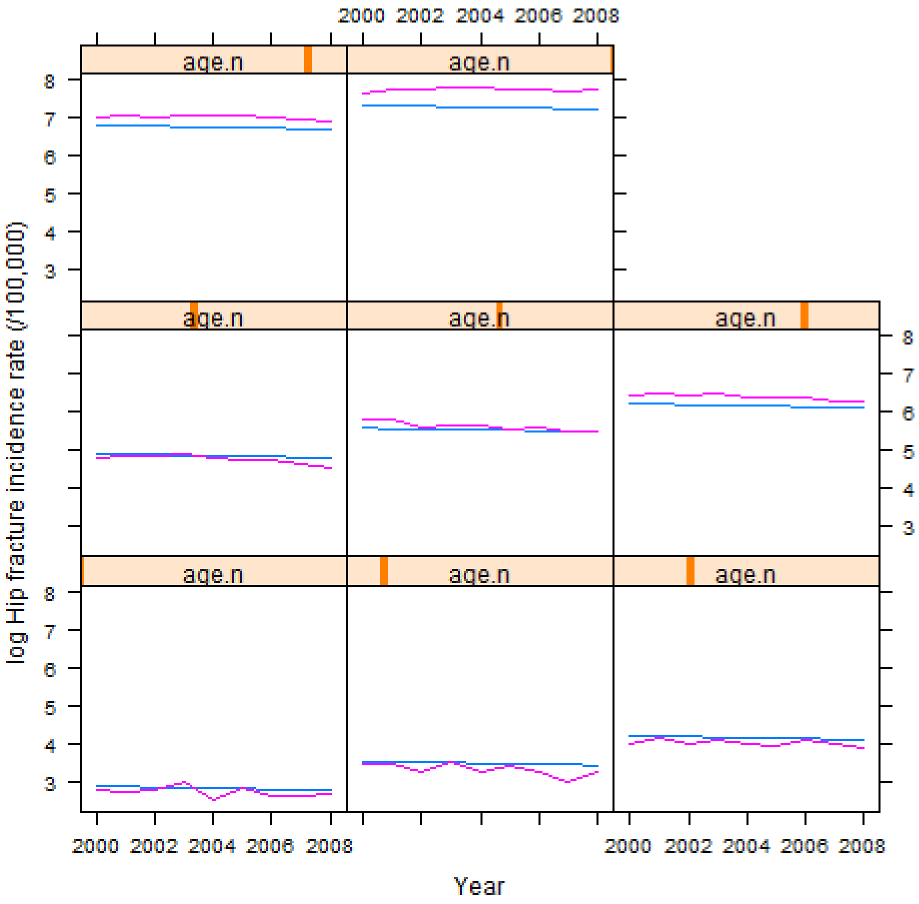

Supplementary figure 20. Observed (red) and predicted (blue) hip fracture incidence rates by age group (from bottom left to top right), Portugal – men

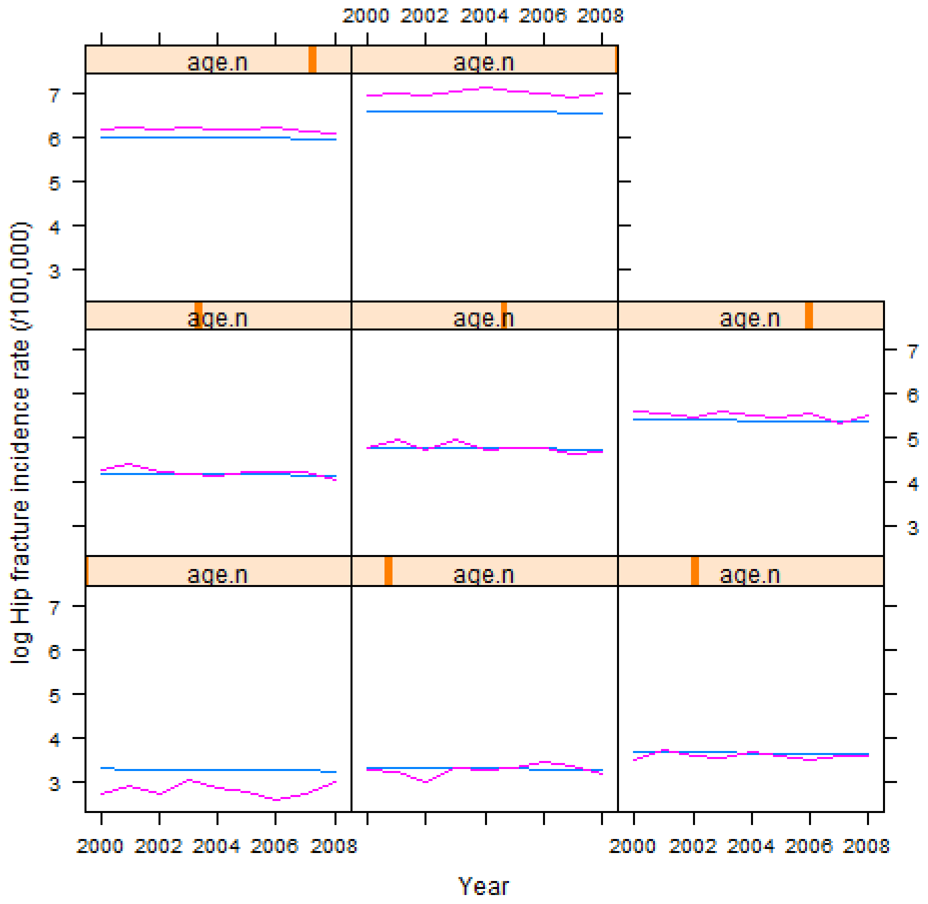

Supplementary figure 21. Observed (red) and predicted (blue) hip fracture incidence rates by age group (from bottom left to top right), Spain – women

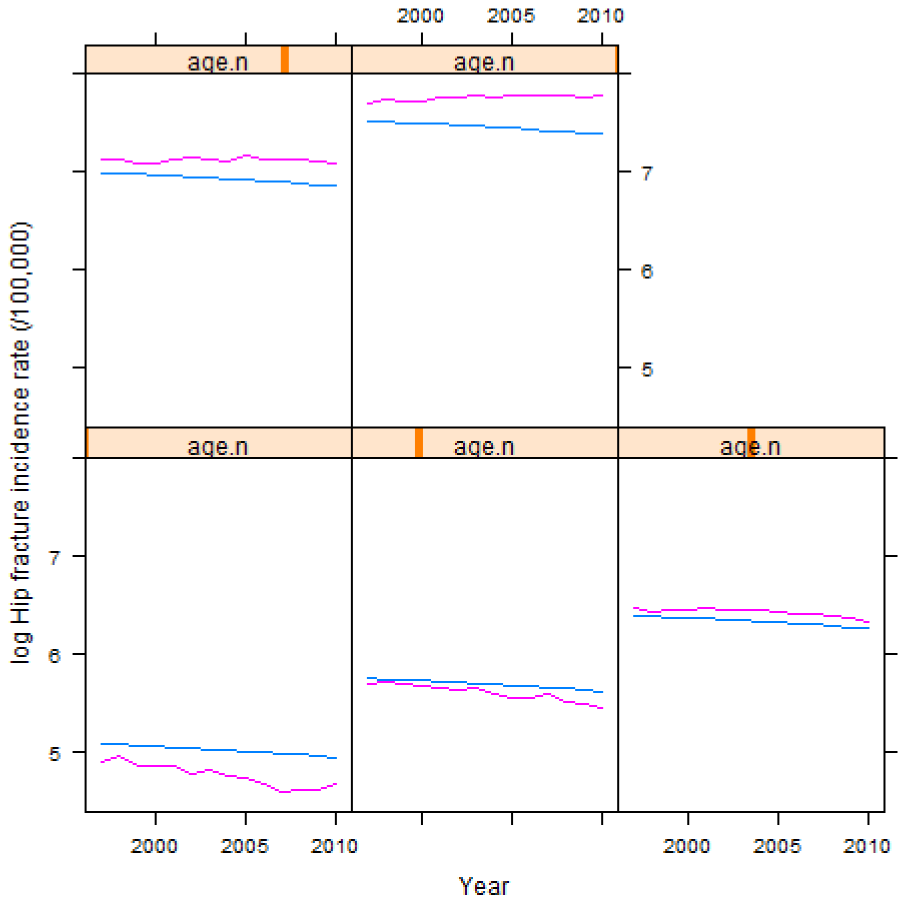

Supplementary figure 22. Observed (red) and predicted (blue) hip fracture incidence rates by age group (from bottom left to top right), Spain – men

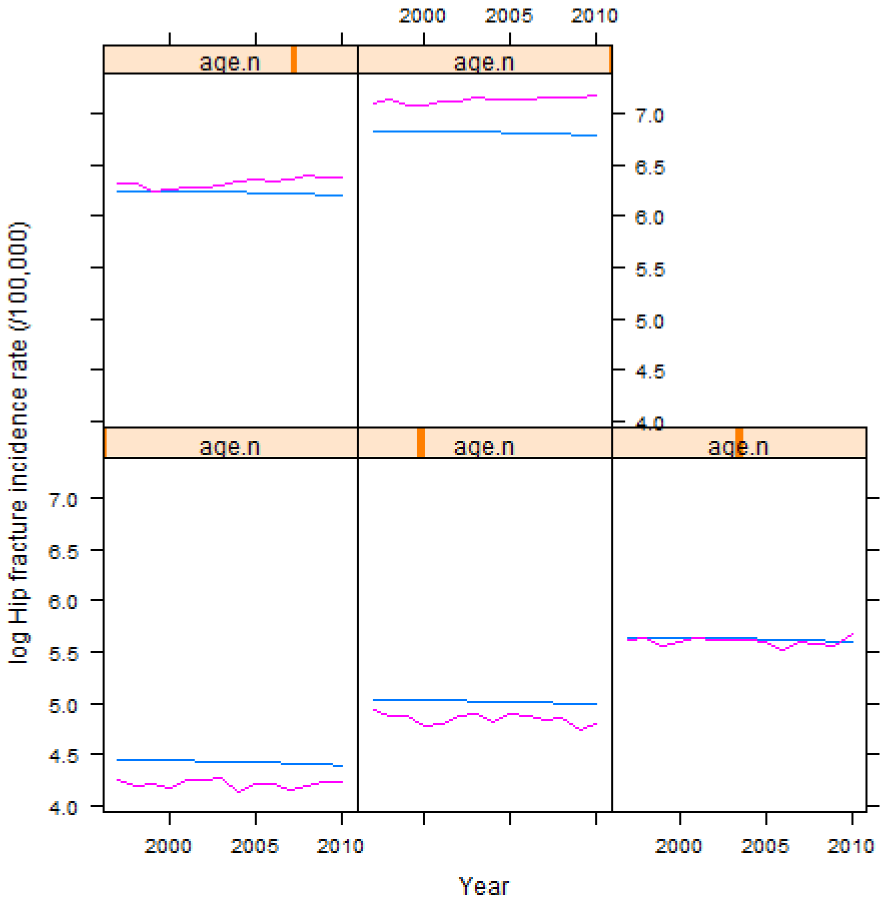

Supplementary figure 23. Observed (red) and predicted (blue) hip fracture incidence rates by age group (from bottom left to top right), Sweden – women

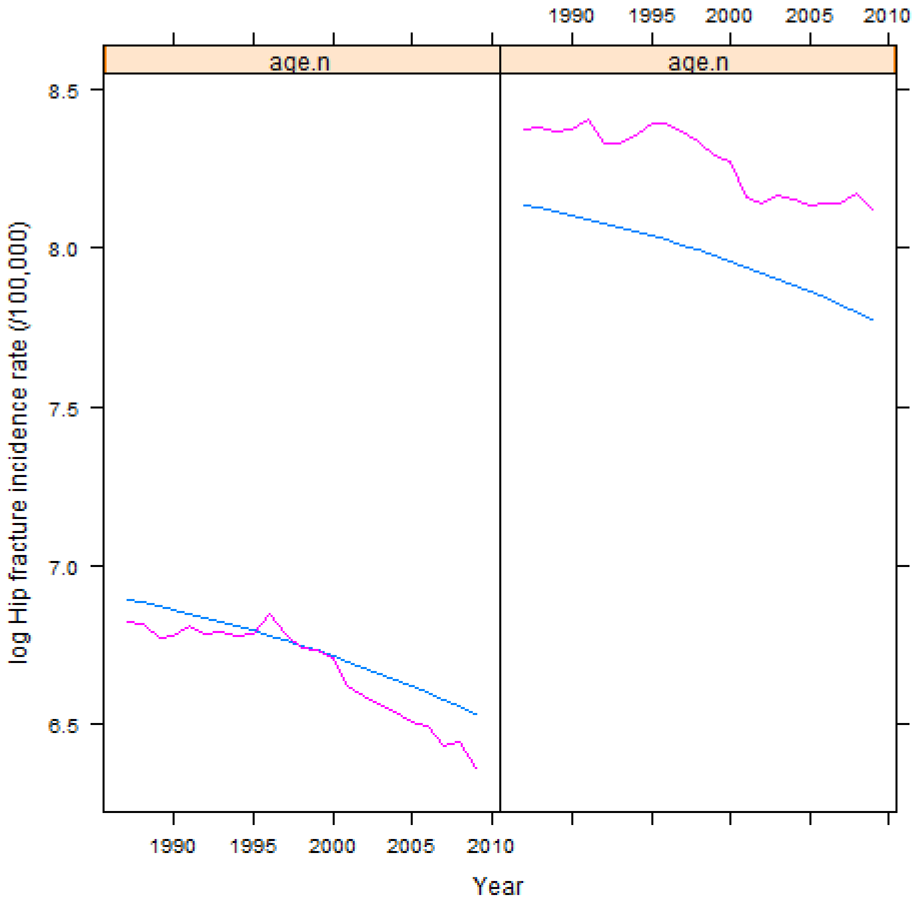

Supplementary figure 24. Observed (red) and predicted (blue) hip fracture incidence rates by age group (from bottom left to top right), Sweden – men

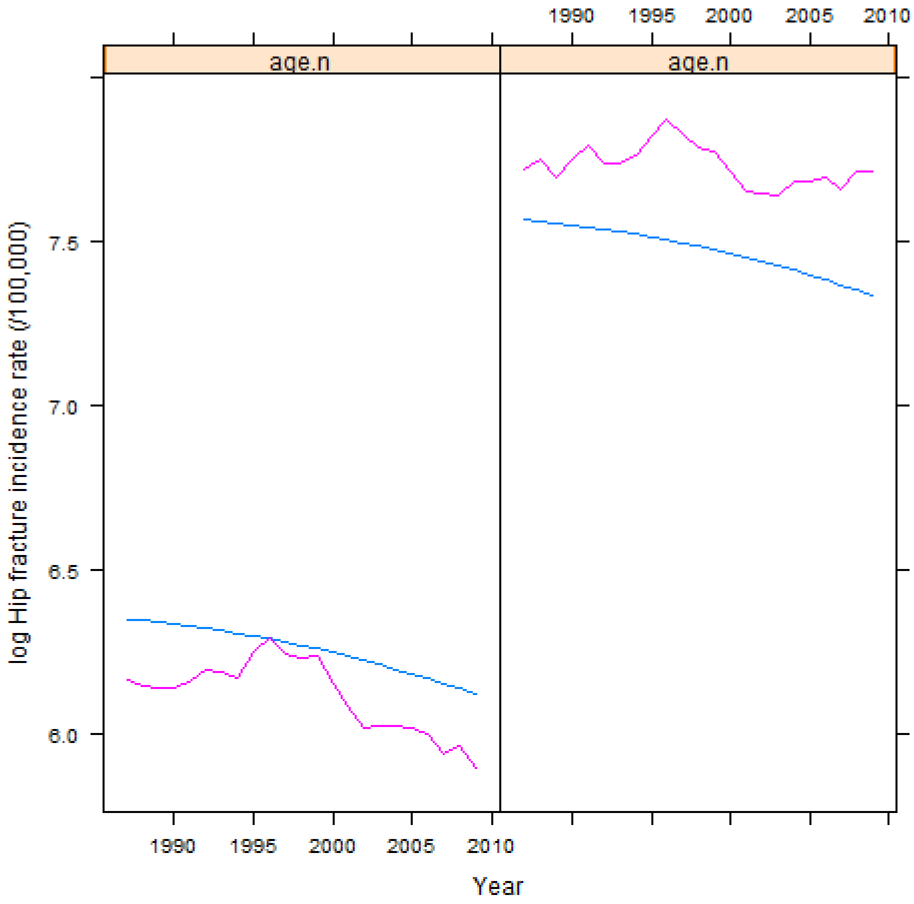

Supplementary figure 25. Observed (red) and predicted (blue) hip fracture incidence rates by age group (from bottom left to top right), Switzerland – women

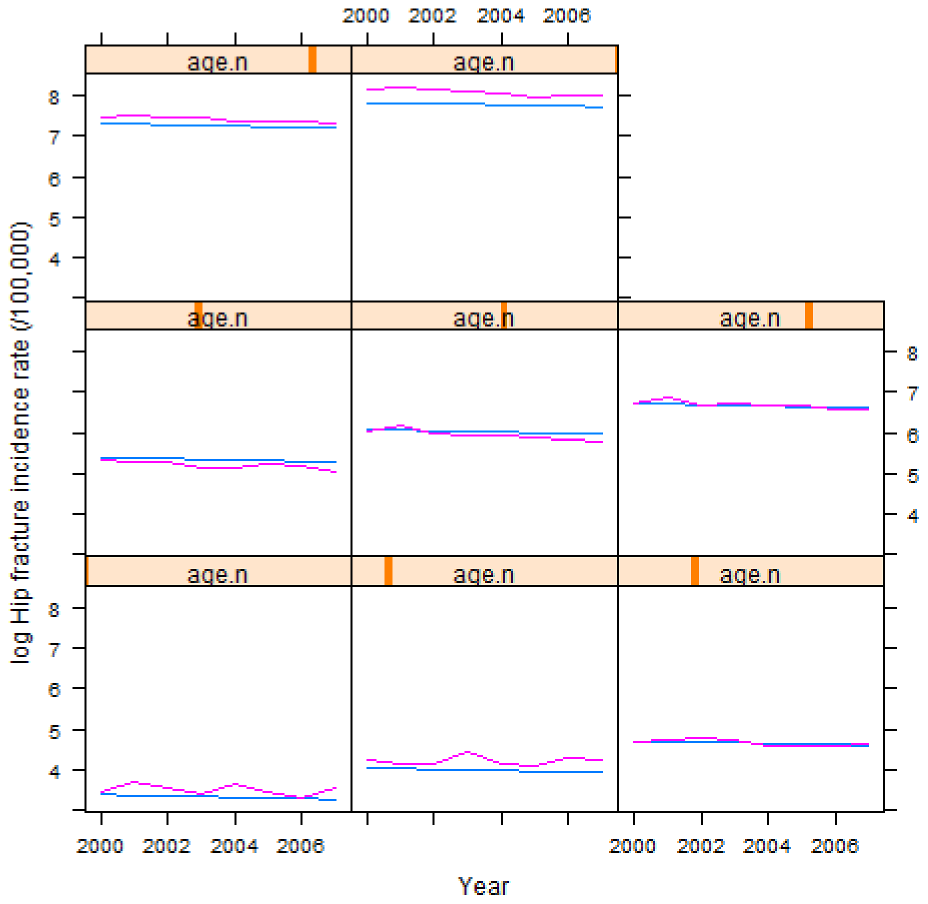

Supplementary figure 26. Observed (red) and predicted (blue) hip fracture incidence rates by age group (from bottom left to top right), Switzerland – men

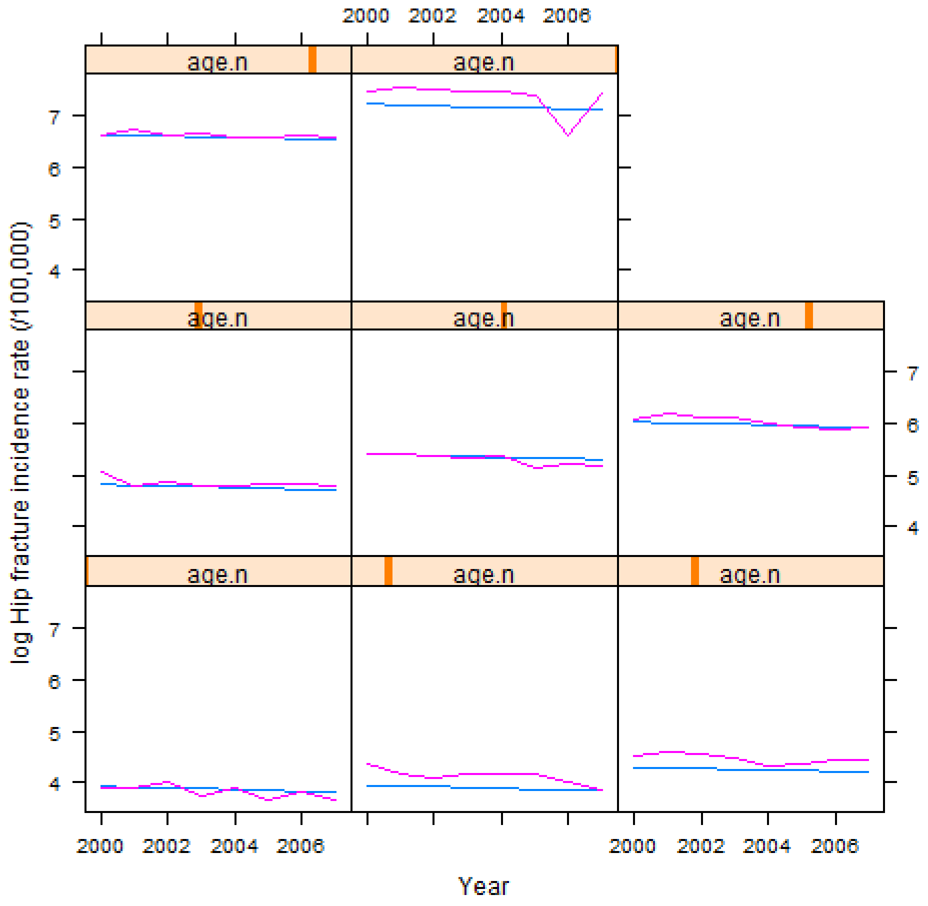

Supplementary figure 27. Observed (red) and predicted (blue) hip fracture incidence rates by age group (from bottom left to top right), UK (England) – women

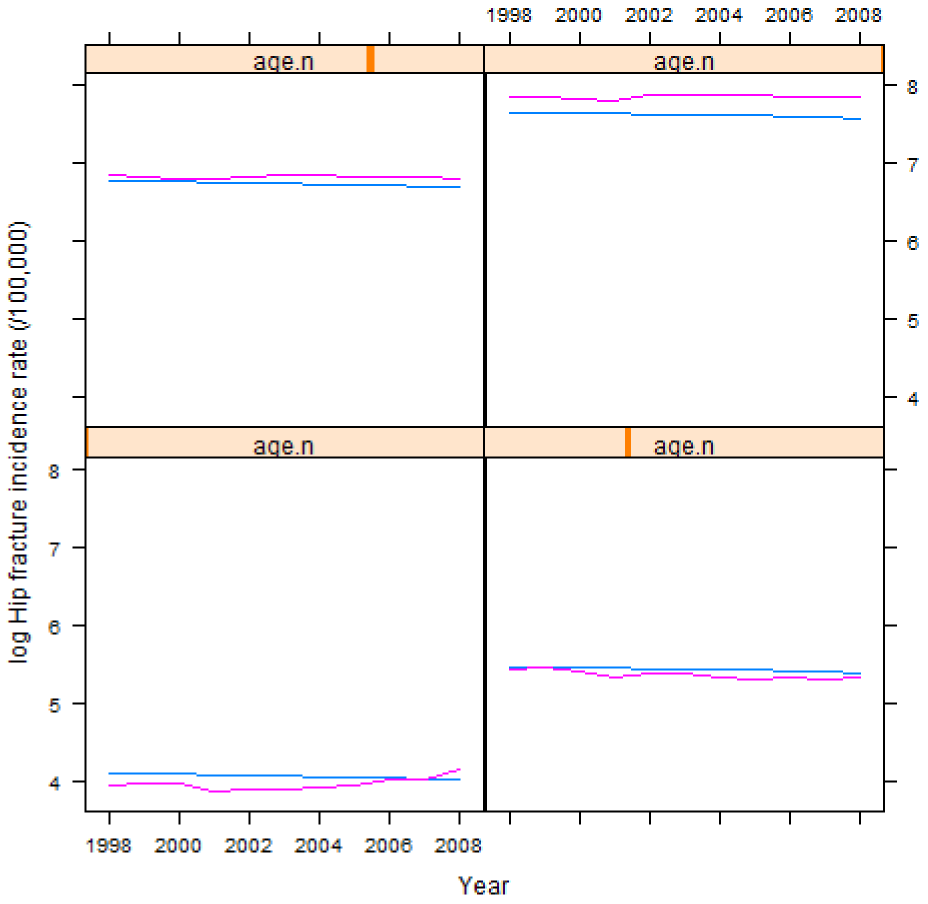

Supplementary figure 28. Observed (red) and predicted (blue) hip fracture incidence rates by age group (from bottom left to top right), UK (England) – men

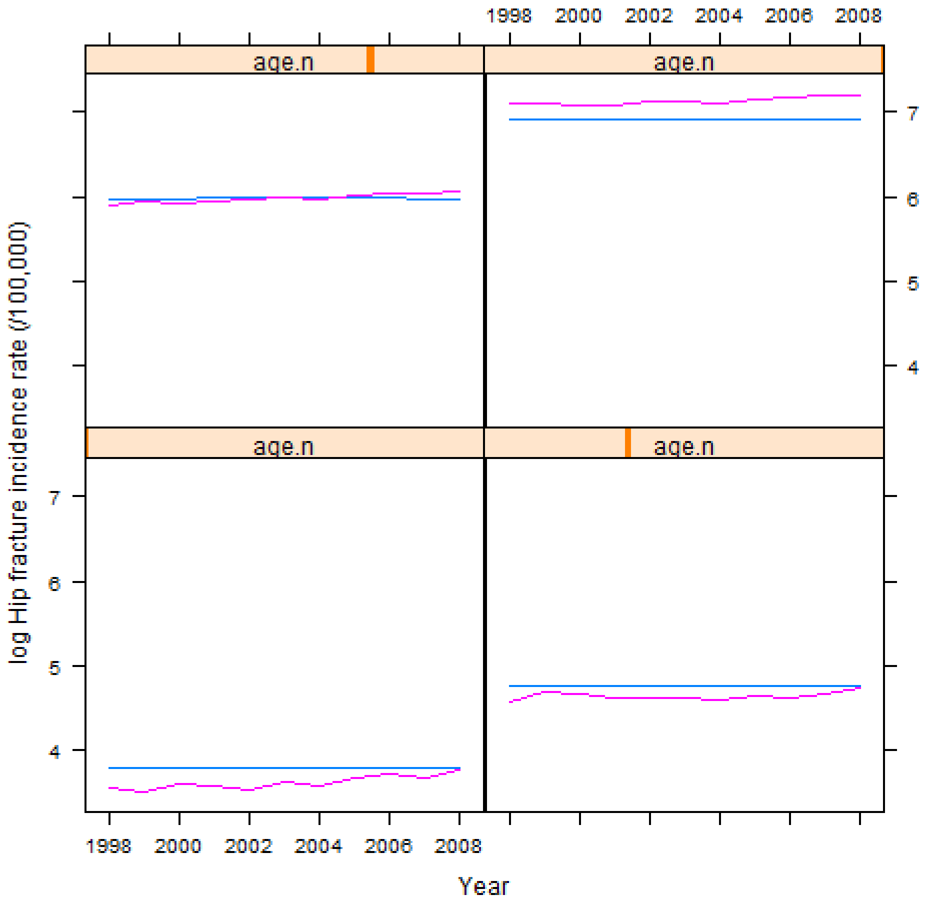

Supplementary figure 29. Bayesian Information Criteria for different cluster solutions (women)

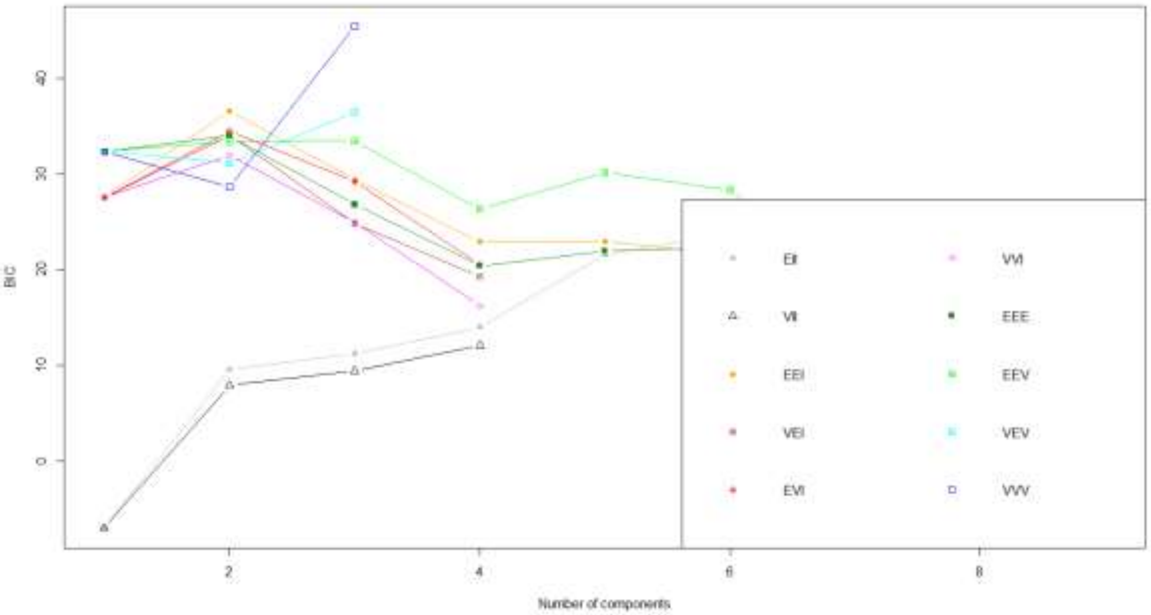

Supplementary figure 30. Bayesian Information Criteria for different cluster solutions (men)

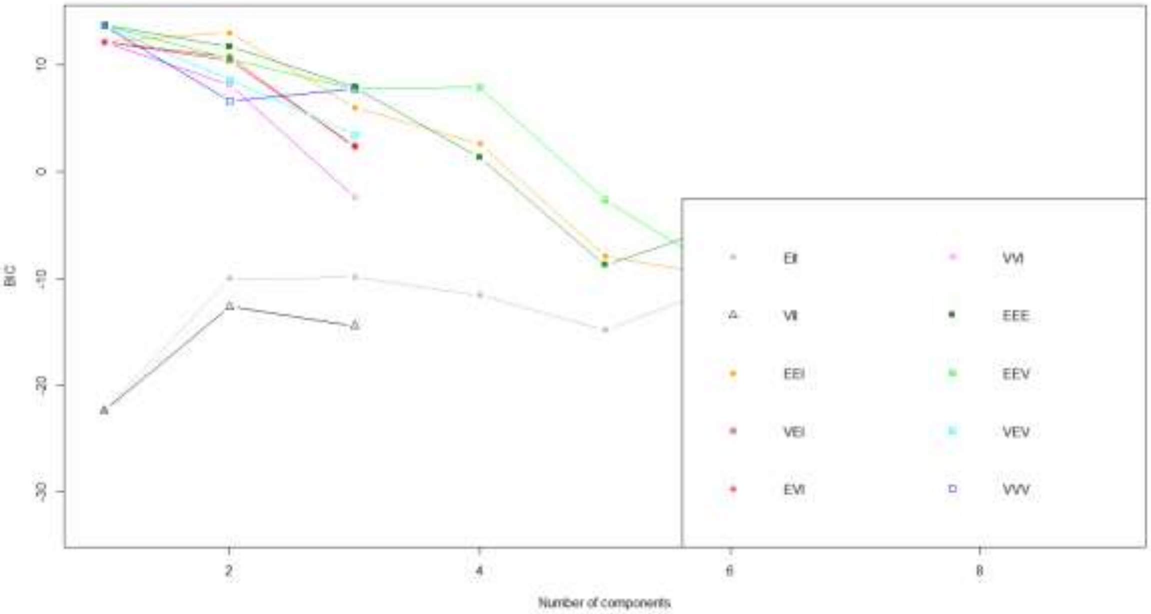

Supplement: Supplementary file 1 — Supplementary materials [file 41598_2017_3847_MOESM1_ESM.pdf]
